# Super-resolving frequency measurement with mode-selective quantum memory

**DOI:** 10.1038/s44460-026-00073-9

**Published:** 2026-05-15

**Authors:** Shicheng Zhang, Aonan Zhang, Ilse Maillette de Buy Wenniger, Paul M. Burdekin, Steven Sagona-Stophel, Anindya Rastogi, Sarah E. Thomas, Ian A. Walmsley

**Affiliations:** 1 Department of Physics, Imperial College London, London, UK; 2 Clarendon Laboratory, University of Oxford, Oxford, UK; 3 Okinawa Institute of Science and Technology, Okinawa, Japan; 4 Department of Engineering Science, University of Oxford, Oxford, UK

**Keywords:** Physics, Optical metrology, Optical sensors, Optical spectroscopy

## Abstract

High-precision optical frequency measurement underpins modern science and technology, yet conventional spectroscopic techniques struggle to resolve sublinewidth spectral features. Here we introduce a platform for super-resolved frequency estimation based on a mode-selective atomic Raman quantum memory implemented in warm caesium vapour. By precisely engineering the light–matter interaction, the memory coherently stores the optimal temporal mode with high fidelity and retrieves it on demand, achieving mode crosstalk as low as 0.34%. To estimate the separation between two spectral lines, we experimentally measure the mean squared error of the frequency estimate, reaching a sensitivity of 1/20 of the linewidth and a (34 ± 4)-fold enhancement in precision over direct intensity measurements. This enhanced frequency resolution, combined with on-demand storage, retrieval and mode-conversion capabilities, establishes a pathway towards multifunctional memory-based time–frequency sensors and their integration within quantum networks.

## Abstract (web summary)

A mode-selective Raman quantum memory in warm caesium vapour enables super-resolved optical frequency estimation with a 34-fold precision enhancement beyond direct intensity measurements.

## Main

The time–frequency (TF) degree of freedom of light underpins applications spanning high-resolution spectroscopy, precision timekeeping^1,2^, ultrafast optics^3^ and emerging quantum technologies^4–7^. In spectroscopy, for example, resolving spectral lines is essential for probing the atomic and molecular properties of matter. Frequency measurements are also crucial for quantum metrology and sensing^8,9^, as they largely rely on measuring the transition between quantized energy levels. However, the precision of these optical measurements has long been constrained by instrumental limitations. Every spectral feature has an intrinsic linewidth set by the Fourier limit—a lower limit being the inverse of the measurement time or the signal’s temporal duration. The resolving power of conventional spectrometers is compounded by the Rayleigh criterion^10^, which defines the minimal resolvable separation between two spectral lines. As the separation shrinks, the uncertainty in their resolution escalates rapidly. This phenomenon, often termed Rayleigh’s curse, poses a critical barrier, particularly when probing weak, photon-limited signals.

The ultimate precision in resolving spectral features is bounded by the quantum mechanical nature of the optical field. From quantum estimation theory, Tsang, Nair and Lu^11^ have shown that Rayleigh’s criterion is not a fundamental limit but rather an artefact of the direct intensity (DI) measurement strategies. Instead, coherent measurements can resolve arbitrarily small separations with constant, finite precision. To circumvent the classical resolution limit, implementing a coherent mode filter to select the optimal mode basis before detection, instead of DI measurements, is essential^12–17^. However, the practical realization of this advantage relies critically on the accuracy of mode filtering, as experimental imperfections such as mode crosstalk and detector noise substantially diminish the achievable precision^18–23^. The high-fidelity coherent mode filtering is generally needed for broader quantum information processing and quantum metrology technologies^5,24–26^. Looking ahead, future quantum networks will demand sensor nodes capable of coherent TF processing, ideally integrated with on-demand buffering for signal synchronization^27,28^, coherent bandwidth and frequency conversion to interface different physical platforms and network channels^29–31^—all while preserving quantum coherence. These integrated functionalities are essential for building robust, distributed quantum-enhanced sensors^32^ capable of dynamically adjusting to varying environmental conditions.

In TF super-resolution, quantum pulse gates, which use nonlinear waveguides for mode-selective frequency conversion, have been deployed to resolve temporal and spectral separations for ultrafast pulses with hundreds-of-GHz bandwidth^33,34^. While quantum pulse gates demonstrate programmable mode selectivity^35^, they inherently lack on-demand storage and buffering capabilities. In the narrowband frequency regime, time–inversion interferometry using gradient echo memory has demonstrated sub-Rayleigh resolution for tens-of-kHz bandwidth pulses^36^, but it is limited to a fixed symmetric–antisymmetric mode selectivity and the ultranarrowband regime, requiring cumbersome magnetic field gradients and cryogenics to map frequency components to longitudinal spatial positions. In addition, there have been works focusing on frequency super-resolution across tens-of-GHz bandwidth using dispersion engineering and electro-optic modulators (EOM)^37,38^. However, no existing platform delivers high-precision super-resolution in the MHz-to-GHz bandwidth together with on-demand storage, retrieval and user-defined mode selectivity.

Here, we introduce a TF super-resolution scheme based on high-fidelity coherent mode filtering in an atomic Raman quantum memory. Photonic quantum memories have been widely studied for absorbing and re-emitting photonic states on demand, with applications in quantum networks, communication and computing^39^. Implemented in warm caesium vapour, our platform utilizes a stimulated Raman process where a strong control field with tailored temporal profile coherently maps an incoming signal field onto a collective atomic spin-wave coherence, achieving mode selectivity up to 99.6% for orthogonal Hermite–Gaussian (HG) temporal modes. Leveraging this user-defined coherent mode filter, we store the optimal signal mode containing the information of spectral line separation, retrieve it on demand and apply maximum-likelihood estimation to the retrieved photon statistics to extract frequency separations. Focusing on sublinewidth frequency separation under various detected photon budgets (from 2 × 10^3^ to 1 × 10^5^), our platform consistently outperforms DI methods, achieving high-precision enhancements. Operating in the MHz-to-GHz bandwidth, our memory-based platform extends the toolbox of TF metrology and provides integrated functionalities encompassing mode filtering, buffering and shaping. These capabilities are ideally suited for next-generation quantum sensor nodes and their deployment in quantum networks.

## Super-resolving measurement in frequency domain

In sensing and metrology, resolution and precision are linked to signal bandwidth and measurement time; specifically, frequency resolution scales directly with the signal bandwidth *σ*. We frame the task of resolving two closely spaced spectral features as estimating their frequency separation normalized by the signal bandwidth, *ϵ* = Δ*ω*/*σ*. Here, Δ*ω* denotes the frequency difference between the two spectral lines centred at *ω*_0_ ± Δ*ω*/2. We model the sources as two mutually incoherent emitters of equal intensity, each described by a Gaussian spectral amplitude $\psi (\omega )={(2{{\uppi }}{\sigma }^{2})}^{-1/4}\exp (-{\omega }^{2}/4{\sigma }^{2})$. Conventional spectroscopy measures the power spectrum,

1 $$S(\omega | \epsilon )=\frac{1}{2}\left[{\left|\psi \left(\omega -{\omega }_{0}-\frac{\epsilon \sigma }{2}\right)\right|}^{2}+{\left|\psi \left(\omega -{\omega }_{0}+\frac{\epsilon \sigma }{2}\right)\right|}^{2}\right].$$

The separation *ϵ* is then estimated by fitting this model to the measured spectrum. The amount of information about *ϵ* extractable from the measurement outcomes is quantified by the Fisher information (FI) per detected photon

2 $${{\mathcal{F}}}_{{\rm{D}}{\rm{I}}}(\epsilon )={\int }_{-\infty }^{\infty }{\rm{d}}\omega \frac{1}{S(\omega | \epsilon )}{\left(\frac{\partial S(\omega | \epsilon )}{\partial \epsilon }\right)}^{2},$$

which vanishes in the limit *ϵ* → 0 (ref. ^11^). The precision, quantified by the variance of an unbiased estimator $\widehat{\epsilon }$, is lower bounded by the Cramér–Rao lower bound (CRLB) ${\rm{V}}{\rm{a}}{\rm{r}}(\widehat{\epsilon })\ge 1/[N{\mathcal{F}}(\epsilon )]$, where *N* represents the number of detected photons. As *ϵ* → 0, the two Gaussian lines increasingly overlap and the estimator variance diverges to infinity, making the estimation of arbitrarily small separations infeasible.

An optimal measurement, by contrast, projects the signal onto an orthonormal HG mode basis with coherent filtering. This mode basis exhibits a constant FI of ${{\mathcal{F}}}_{{\rm{H}}{\rm{G}}}(\epsilon )\approx 1/4$ that saturates the quantum FI—the ultimate limit of precision attainable over all possible measurements for a given input quantum state^11^. As the HG measurement is particularly advantageous for small separations, we will focus on the precision and ability to resolve small separations under limited photon budgets in realistic experiments. For small separations, the projection onto the HG_0_ and HG_1_ mode contributes most of the FI. The ideal projection probabilities onto HG_0_ and HG_1_ modes define a raw estimator ${\widehat{\epsilon }}_{{\rm{r}}{\rm{a}}{\rm{w}}}=4\sqrt{{N}_{1}/{N}_{0}}$, where *N*_0_ and *N*_1_ are the experimentally measured counts of HG_0_ and HG_1_ projections.

In practical implementations, mode crosstalk modifies the ideal projection probabilities. We can model this effect between the first two HG modes by describing how the ideal probability vector, **p**, is perturbed into the measured probability vector, $\widetilde{{\bf{p}}}$. This transformation is defined by a coupling matrix *M*, as

3 $$\widetilde{{\bf{p}}}=M{\bf{p}}=\left(\begin{array}{ll}\alpha & 1-\beta \\ 1-\alpha & \beta \end{array}\right)\left(\begin{array}{l}P(0| \epsilon )\\ P(1| \epsilon )\end{array}\right),$$

where *α*, *β* ∈ [0, 1] quantify the mode filtering fidelity. The resulting perturbed probability $$\widetilde{{\bf{p}}}=(\begin{array}{l}\widetilde{P}(0| \epsilon )\\ \widetilde{P}(1| \epsilon )\end{array})$$ is then normalized. Assuming low mode crosstalk *α* ≈ 1 and *β* ≈ 1, we derive the FI for small separations as (see Supplementary Section 1 for details)

4 $${\mathcal{F}}(\epsilon )\approx \frac{1}{4[(1-\alpha )/{(\epsilon /4)}^{2}+1]}.$$

The FI in the presence of mode crosstalk is highly sensitive to the leakage from the HG_0_ mode into the HG_1_ mode, 1 − *α*. If (*ϵ*/4)^2^ ≪ 1 − *α*, the FI decreases significantly from the ideal case, highlighting the importance of low-crosstalk mode filtering in resolving very small separations.

To estimate *ϵ* in the presence of crosstalk, we use maximum likelihood estimation (MLE) based on the perturbed projection probabilities onto the first two HG modes, $\widetilde{P}(0| \epsilon )$ and $\widetilde{P}(1| \epsilon )$. The MLE finds the parameter value that maximizes the likelihood function ${\mathcal{L}}({\bf{x}}| \epsilon )={\prod }_{i=1}^{N}\widetilde{P}({x}_{{i}}| \epsilon )$ for observing the measured data **x** = {*x*_1_, *x*_2_, …, *x*_*N*_}. For small separations, the MLE estimator can be written as

5 $${\hat{\epsilon }}_{{\rm{M}}{\rm{L}}{\rm{E}}}=\mathop{{\rm{a}}{\rm{r}}{\rm{g}}{\rm{m}}{\rm{a}}{\rm{x}}}\limits_{\epsilon }[{N}_{0}\mathrm{ln}\tilde{P}(0|\epsilon )+{N}_{1}\mathrm{ln}\tilde{P}(1|\epsilon )],$$

subject to the parameter space constraint $\varTheta =\{\epsilon \in {{{\mathbb{R}}}}| \epsilon \ge 0\}$. The MLE is asymptotically unbiased and efficient in the limit *N* → *∞*, implying that its variance approaches the CRLB.

In real experiments with finite statistics, the MLE exhibits a non-zero bias, particularly at small separations. This bias arises from both higher-order asymptotic terms^40^ and the non-negativity parameter space constraint *Θ*. Owing to shot noise, the experimentally observed normalized counts $${\bf{f}}=(\begin{array}{l}{N}_{0}/N\\ {N}_{1}/N\end{array})$$ may lie outside the physically valid range. When this occurs, the likelihood function ${\mathcal{L}}({\bf{x}}| \epsilon )$ takes its maximum at the boundary of the parameter space constraint (that is *ϵ* = 0), leading to a positively skewed distribution of the estimator. This skewing effect results in an unavoidable bias for *ϵ* → 0, with the magnitude of the bias determined by the estimator’s distribution. The bias diminishes with higher photon counts *N*, as statistical fluctuations on *N*_0_ and *N*_1_ decrease, making the estimator’s distribution at the boundary less probable (see Supplementary Section 1 for simulation results).

We assess our measurement performance using the mean squared error (MSE), ${\rm{M}}{\rm{S}}{\rm{E}}(\epsilon ,N)=\langle {(\widehat{\epsilon }-\epsilon )}^{2}\rangle$, incorporating both the variance and the squared bias of the estimator, ${\rm{M}}{\rm{S}}{\rm{E}}(\epsilon ,N)={\rm{V}}{\rm{a}}{\rm{r}}(\widehat{\epsilon })+b{(\epsilon ,N)}^{2}$. The MSE is lower bounded by the CRLB with a bias term^41^

6 $${\rm{M}}{\rm{S}}{\rm{E}}(\epsilon ,N\,)\ge \frac{{[1+{b}^{{\prime} }(\epsilon ,N\,)]}^{2}}{N{\mathcal{F}}(\epsilon )}+b{(\epsilon ,N\,)}^{2},$$

where ${b}^{{\prime} }(\epsilon ,N)$ represents the derivative of *b*(*ϵ*, *N*) with respect to *ϵ*. Only as *b*(*ϵ*, *N*) → 0 does the MSE converge to the standard unbiased CRLB, ${\rm{V}}{\rm{a}}{\rm{r}}(\widehat{\epsilon })\ge 1/[N{\mathcal{F}}(\epsilon )]$. From the MSE, we quantify the sensitivity of the apparatus as the minimal resolvable separation ${\epsilon }_{\min }$ for which the parameter-to-error ratio (PER) of the estimate,

7 $${\rm{P}}{\rm{E}}{\rm{R}}(\epsilon ,N\,)={\epsilon }^{2}/{\rm{M}}{\rm{S}}{\rm{E}}(\epsilon ,N\,),$$

is greater than 1 (ref. ^19^). This minimal resolvable separation defines a modified Rayleigh criterion for resolving spectral lines under certain detected photon budgets. In this work, MSE and PER serve as the figures of merit to quantify the performance of our experimental platform.

## Mode-selective Raman quantum memory

**Fig. 1:**
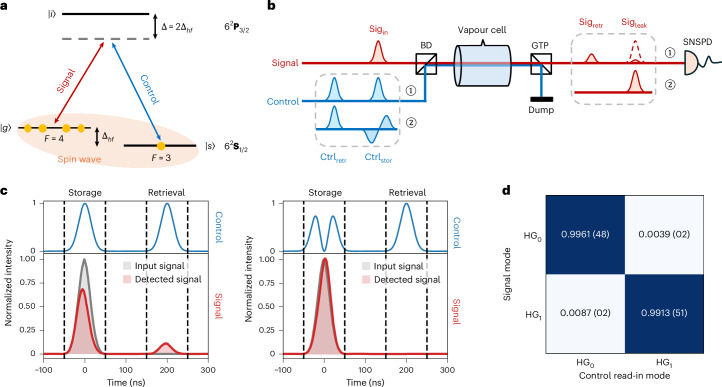
Mode-selective Raman quantum memory. **a**, Raman memory energy levels. The states $| g\rangle$ and $| s\rangle$ are the hyperfine levels of the atomic ground state, separated by an energy splitting of Δ_*hf*_ = 9.2 GHz. Atoms are initially prepared in state $| g\rangle$. A weak signal field and a strong control field coherently map the incoming signal onto a collective atomic spin-wave coherence stored between $| g\rangle$ and $| s\rangle$. **b**, Schematic of a Raman memory as a coherent temporal mode filter. The signal and control fields copropagate through a vapour cell after being combined by a BD. Following interaction, the signal is detected by SNSPDs, while the control field is suppressed by a GTP and a series of etalons. When both the input signal Sig_in_ and the control read-in pulse Ctrl_stor_ are in the HG_0_ mode, storage occurs (the leaked signal, Sig_leak_, is weaker than Sig_in_), followed by successful retrieval (Sig_retr_) using the control read-out pulse (Ctrl_retr_) (labelled as 1). By contrast, when Sig_in_ is HG_0_ and Ctrl_stor_ is HG_1_, minimal storage or retrieval is observed, demonstrating mode selectivity (labelled as 2). **c**, Examples of experimentally detected signal sequences for the HG_0_ signal stored using HG_0_ and HG_1_ control modes, respectively. **d**, Measured mode crosstalk matrix. The matrix elements are calculated from the corresponding total efficiencies, normalized across each row. The uncertainties assume Poissonian shot noise.

To achieve frequency-domain super-resolution, we deploy a Raman quantum memory to perform coherent temporal mode filtering. The Raman memory operates within a warm vapour ensemble of caesium-133 atoms, featuring a Λ-type three-level system as shown in Fig. 1a. We select the two hyperfine ground states, $| F=4\rangle$ and $| F=3\rangle$ of the 6^2^**S**_1/2_ manifold, as the initial state $| g\rangle$ and storage states $| s\rangle$, respectively, and use the 6^2^**P**_3/2_ level as the intermediate excited state $| i\rangle$. A strong classical control field (coupled to the $| s\rangle \leftrightarrow | i\rangle$ transition with a detuning Δ) coherently maps a weak signal field (coupled to the $| g\rangle \leftrightarrow | i\rangle$ transition with the same detuning) into a collective atomic coherence (spin wave) between $| g\rangle$ and $| s\rangle$. As shown in Fig. 1b, the orthogonally polarized signal and control fields are combined using a beam displacer (BD) and copropagate through the vapour cell. The read-in control pulse Ctrl_stor_ stores the signal pulse Sig_in_ as a spin wave, while unabsorbed light exits as the leaked signal Sig_leak_. Retrieval is performed on demand by a second control pulse, Ctrl_retr_, which converts the stored excitation back into an optical field, Sig_retr_. By shaping the temporal profile of Ctrl_retr_, we retrieve the signal into a user-defined temporal mode, which may differ from the original read-in mode. After the memory, the control field is suppressed using a Glan–Taylor polarizer (GTP) and three double-passed Fabry–Pérot etalons, leaving only the retrieved signal for detection via superconducting nanowire single-photon detectors (SNSPDs).

**Extended Data Fig. 1:**
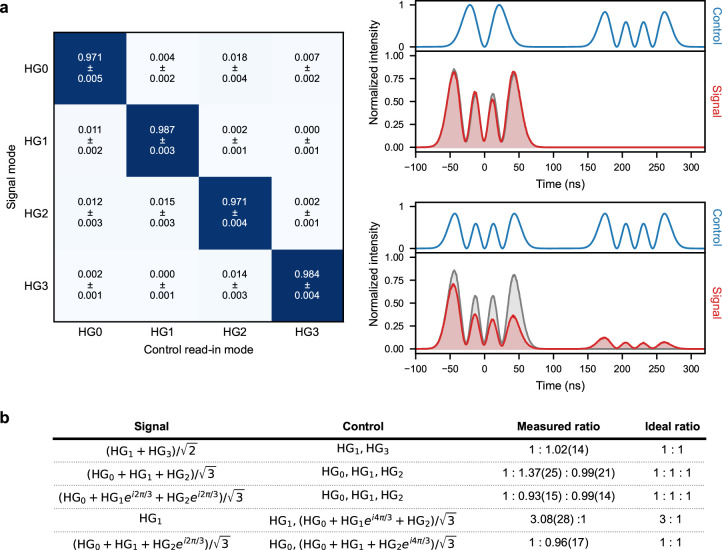
Programmability of the temporal mode filter. (**a**) Measured mode crosstalk matrix for the first four HG modes. Matrix elements are calculated from the corresponding total efficiencies, normalized across each row. The right panels display example experimental traces of an HG3 signal stored using HG1 (top) and HG3 (bottom) control fields, respectively, and retrieved using an HG1 control. (**b**) Mode filtering of temporal mode superpositions. The measured ratios are derived from the corresponding storage efficiencies.

In the low-coupling regime (that is, at low storage efficiencies), the memory operates as a fully programmable single-mode device where the stored temporal mode is directly defined by the temporal profile of the control pulse^42^ (see Supplementary Discussin Section 2). Figure 1b shows two representative cases: when both signal and control are in the HG_0_ mode (labelled 1), the signal is efficiently stored and retrieved; conversely, when the signal is in HG_0_ but the control is in HG_1_ (labelled 2), storage and retrieval are strongly suppressed. Experimental results for these scenarios are shown in Fig. 1c. To quantify the mode selectivity, we measured the total efficiencies for all combinations of signal and control pulses prepared in HG_0_ and HG_1_ modes. Figure 1d shows the measured mode crosstalk matrix with high mode selectivity: 99.61% ± 0.48% when storing HG_0_ signals with HG_0_ control, and 0.39% ± 0.02% with HG_1_ control. These measurements used read-in and read-out control pulses of 125 ± 1 pJ, yielding total efficiencies of about 11% along the diagonal. We further demonstrate filtering of higher-order HG modes and their superpositions (Extended Data Fig. 1). At higher coupling strengths (for example, stronger control pulses), this single-mode behaviour degrades owing to increased storage of modes orthogonal to the target control mode. This highlights an inherent trade-off between mode selectivity and overall memory efficiency. By achieving low crosstalk and high mode selectivity in the low-coupling regime, our Raman memory provides a robust and effective platform for the super-resolution task.

## Experimental super-resolution with mode-selective quantum memory

To perform estimation experiments using the mode-selective Raman quantum memory described above, we prepared the signal as an incoherent mixture of two Gaussian spectral lines, each with linewidth *σ* = 5.30 MHz, and varied their normalized separation from 0 to 1 in steps of 0.05 (Methods). For each separation, we performed two measurements by storing the signal with either an HG_0_ or HG_1_ control read-in pulse. In both cases, the stored signals were retrieved after 200 ns using an HG_1_ pulse and detected with SNSPDs. At *ϵ* = 0, the storage and total internal efficiencies for HG_0_ read-in were 39.5% ± 0.6% and 14.6% ± 0.1%, respectively, using control pulses of 130 ± 1 pJ. For each separation, we recorded approximately *N* ≈ 2 × 10^5^ detector clicks in total.

To estimate the frequency separation *ϵ*, we first calibrated the system with 1.6 × 10^5^ detected counts per separation. By fitting the experimental model to this calibration data, we determined the measured crosstalk from HG_0_ to HG_1_ in the estimation experiment to be 0.34%. This calibration was performed in a separate experiment from the previous mode-filtering demonstration, accounting for the slight difference in reported crosstalk. With the calibrated model, in each run, we performed MLE to compute the estimate ${\widehat{\epsilon }}_{{\rm{M}}{\rm{L}}{\rm{E}}}$ on the basis of equation (5) with registered counts (*N*_0_, *N*_1_). We then obtained the MSE for each separation by averaging ${({\widehat{\epsilon }}_{{\rm{M}}{\rm{L}}{\rm{E}}}-\epsilon )}^{2}$ over 50 bootstrapped experiments with the same *N* drawn from the full click dataset. The uncertainty in the MSE was evaluated by repeating this procedure ten times.

**Fig. 2:**
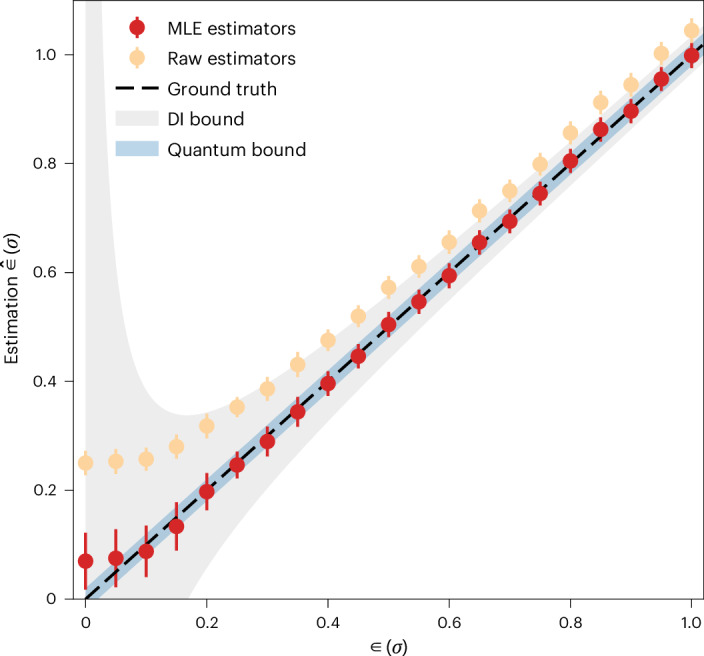
Experimental estimation results. MLEs (red) with raw estimators (yellow), both derived from 10^4^ total detected photons. The markers and error bars denote the means and standard deviations derived from 50 bootstrap resamples. The dashed black line indicates the ground truth separation. The shaded regions illustrate the theoretically predicted standard deviations from the quantum CRLB (blue) and the DI measurement CRLB (grey).

**Extended Data Fig. 2:**
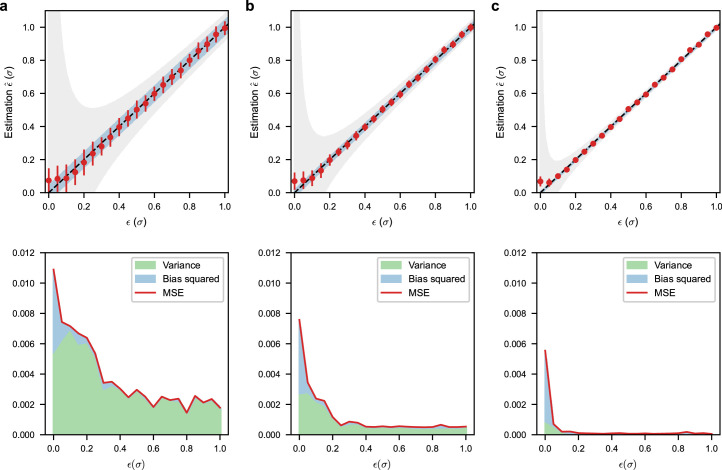
Maximum likelihood estimation results for different total detection counts. Maximum likelihood estimation results for total detection counts of (**a**) 2 × 10^3^, (**b**) 10 × 10^3^, and (**c**) 100 × 10^3^. The upper panels show the maximum likelihood estimators, along with the corresponding quantum bounds (blue region) and direct intensity measurement bounds (gray region). Red dots and error bars denote the means and standard deviations derived from 50 bootstrap resamples. The lower panels display the MSE (red line), with contributions from bias squared (blue region) and variance (green region).

Figure 2 shows the estimation results with estimator standard deviations for *N* = 10 × 10^3^ detected counts. The complete estimation results for various detection counts, detailing the contributions from estimator bias and variance, are shown in Extended Data Fig. 2. While the raw estimators $4\sqrt{{N}_{1}/{N}_{0}}$ have low variance, they suffer from a systematic bias caused by mode crosstalk and control-field leakage. By contrast, the MLE exhibits substantially reduced bias. For the smallest separations (*ϵ* = 0 − 0.2), the MLE estimators display lower bias but higher variance, reflecting the trade-off between estimator variance (precision) and bias (accuracy) described in equation (6). Crucially, all MLE error bars are smaller than the DI bound, demonstrating enhanced precision over DI detection. To validate the platform’s unique functionality of on-demand storage, retrieval and coherent mode conversion, we also performed super-resolving estimation with storage times from 150 ns to 250 ns and with HG_0_ and HG_1_ retrieval modes. The results are included in Supplementary Section 3.

**Fig. 3:**
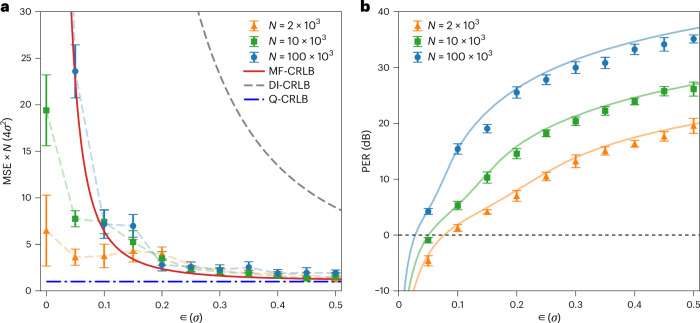
Estimation performance under different photon budgets. **a**, MSE scaled by photon number *N*. Experimental results for *N* = 2 × 10^3^ (orange triangles), 10 × 10^3^ (green squares) and 100 × 10^3^ (blue circles) approach the theoretical CRLB of our set-up (solid red line) as *N* increases. The quantum CRLB (dash-dotted blue line) and the DI detection CRLB (dashed grey line) are shown for reference. **b**, PER for different detection counts. Experimental data (markers) are plotted alongside their corresponding theoretical values (solid lines). The dashed line at 0 dB marks the sensitivity threshold. The markers and error bars denote the means and standard deviations derived from ten bootstrap resamples for both plots.

The residual MLE bias near *ϵ* = 0 is mainly attributed to the non-negativity constraint on *ϵ*. This bias is unavoidable under limited photon budgets and depends on the number of detected photons. Figure 3a shows MSE × *N* together with the corresponding CRLBs for comparison across different detected counts. For *ϵ* < 0.5, the measured MSE is generally smaller than the DI CRLB, indicating improved precision. However, at low photon numbers (for example, *N* = 2 × 10^3^), the MSE falls below the mode-filtering CRLB for *ϵ* < 0.15 owing to estimation bias. As *N* increases to 100 × 10^3^, the MSE approaches the CRLB as the bias decreases, consistent with earlier discussion. Thus, while the MLE introduces a small bias at low photon numbers and small separations, its contribution to the MSE becomes negligible at larger *N*, enabling reliable high-precision estimation.

## Sensitivity and precision enhancement benchmarking

To benchmark the sensitivity of our measurement scheme, we examine the minimum resolvable separation using the PER defined in equation (7). Figure 3b shows PER as a function of separation for different total detection counts. With 100 × 10^3^ detected photons, we achieve PER = 4.4 ± 0.5 dB at *ϵ* = 0.05, corresponding to resolving spectral lines separated by just 265 kHz for a linewidth of 5.30 MHz. By contrast, for lower photon numbers (*N* = 2 × 10^3^ and 10 × 10^3^), PER < 0 dB at *ϵ* = 0.05, indicating that such small separations cannot be distinguished from zero separation at these photon levels. The theoretical PER values (Fig. 3b, solid lines) agree well with the experimental results.

In addition to sensitivity, we evaluate the precision enhancement enabled by our memory-based mode filtering scheme against DI methods. We quantify the enhancement via the super-resolution parameter ${\mathfrak{s}}$ introduced by Mazelanik et al.^36^, defined as the ratio of the FI for the two methods in the limit *ϵ* → 0,

8 $${\mathfrak{s}}=\mathop{\mathrm{lim}}\limits_{\epsilon \to 0}({\mathcal{F}}/{{\mathcal{F}}}_{{\rm{D}}{\rm{I}}}).$$

**Fig. 4:**
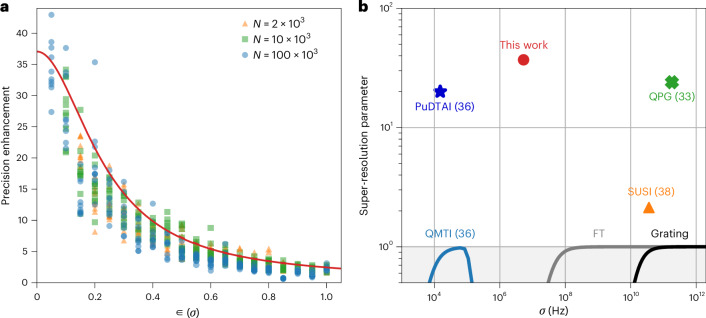
Precision enhancement benchmarking. **a**, Precision enhancement as a function of spectral line separation *ϵ*. The theoretical precision enhancement (red curve) is plotted with all experimental results for various photon counts: *N* = 2 × 10^3^ (orange triangles), 10 × 10^3^ (green squares) and 100 × 10^3^ (blue circles). **b**, Comparison of super-resolution parameters for various schemes. This panel benchmarks the performance of our system against other super-resolution techniques and DI methods. FT, Fourier transform spectrometer (Bruker IFS 125HR); Grating, a grating spectrometer of 1,200 lines per mm; PuDTAI, pulse-division time-axis-inversion^36^; QMTI, quantum-memory temporal imaging^36^; QPG, quantum pulse gate^33^; SUSI, super-resolution via spectral inversion^38^.

This parameter characterizes the achievable precision enhancement in the infinite photon limit, where a truly unbiased estimator exists only as *N* → *∞*. This parameter can be used to assess and compare the performance of various super-resolving measurement schemes, with its value primarily determined by mode crosstalk. Figure 4a shows how the theoretical enhancement ${\mathcal{F}}/{{\mathcal{F}}}_{{\rm{D}}{\rm{I}}}$ (red curve) varies with *ϵ*. In the limit *ϵ* → 0, the ratio asymptotically approaches 37 for our platform. This value sets a new benchmark for frequency-separation estimation, surpassing previous results^33,36,38^, as shown in Fig. 4b. For comparison, DI methods—including quantum-memory temporal imaging^36^, Fourier transform spectroscopy and grating spectroscopy—all yield ${\mathfrak{s}} \leq 1$. Among techniques spanning various bandwidth regimes, our Raman memory uniquely operates in the MHz-to-GHz range, set by the hyperfine splitting and the memory storage time. For a comprehensive comparison, see Supplementary Section 4.

Under finite statistics, the experimental precision enhancement can deviate from the theoretical values owing to estimator bias. In practice, when the bias is negligible, the enhancement can be approximated by the ratio of the measured mode-filtering MSE to the CRLB of DI methods. Shown as data points in Fig. 4a, these ratios follow the trend of the theoretical prediction, subject to experimental imperfections and fluctuations. However, at very small separations, the estimator bias can cause the measured MSE to fall below the CRLB, resulting in calculated ratios exceeding theoretical values. In this region, both the mode filtering method and the DI method exhibit bias, making the determination of realistic precision enhancement more complex^18^. Our experiment demonstrates a practical 34 ± 4-fold improvement at *ϵ* = 0.05 and a 28 ± 6-fold improvement at *ϵ* = 0.1 using 100 × 10^3^ detected photons, confirming the feasibility and effectiveness of our approach. Furthermore, we expect that DI measurements, which inherently have larger estimator variances from the FI analysis, require a higher number of photons to achieve nearly unbiased estimation, whereas the optimal mode filtering maintains lower variance even in photon-limited applications.

## Conclusion

We have demonstrated that all-optical control of Raman interactions enables a compact, room-temperature quantum memory to function as a programmable, high-precision TF sensor across the MHz-to-GHz regime. Beyond resolving two spectral lines, our platform’s fully programmable temporal mode filtering allows us to resolve more complex spectral structures. This flexibility is essential for multiparameter estimation, where the optimal measurement basis can be complicated depending on the task^43–45^; such measurements could be implemented via TF mode sorting using cascaded memories or loop architectures. Overall, the combination of high-fidelity mode filtering, on-demand storage and mode conversion establishes this platform as a versatile tool for advanced TF metrology and photon-limited sensing.

The technology developed here holds promise for diverse applications, including high-precision clock synchronization^46^, spacetime positioning^47,48^, photon dose-limited metrology^6^ and TF-encoded quantum information processing^5,24,26^. For frequency metrology, our broadband, mode-selective measurement enables high-precision sensing in regimes where traditional spectroscopic techniques become impractical. In Doppler LiDAR, which infers target velocity from frequency shifts of backscattered light, conventional techniques fall into two classes: heterodyne detection which imposes stringent phase coherence requirements, and incoherent edge techniques using narrowband filters, which suffer from a trade-off between sensitivity and dynamic range. Our platform bypasses these bottlenecks by providing programmable, high-resolution measurements of frequency shifts. Our current demonstration (5.30 MHz bandwidth at 852 nm) resolves frequency shifts down to 265 kHz, which corresponds to a velocity resolution of 0.11 m s^−1^ and a range resolution of 38 cm at MHz measurement rates, even under low signal-to-noise ratio conditions. Furthermore, the platform’s flexibility permits complex tasks such as resolving multiple proximal targets or the joint estimation of velocity and range^49,50^ (see Supplementary Section 5 for more discussions).

**Fig. 5:**
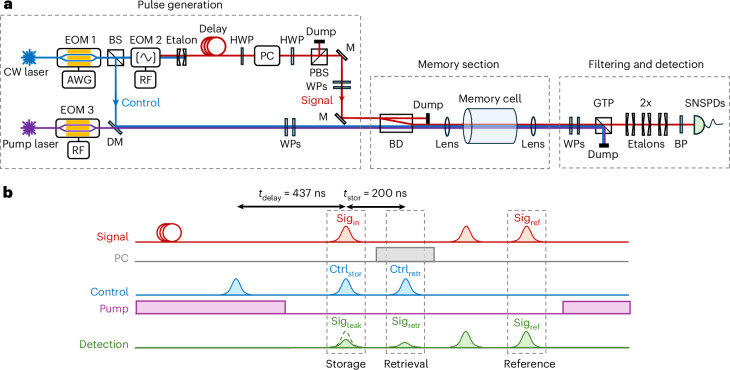
Schematic of experimental set-up. **a**, Experimental set-up. The apparatus consists of three main sections: pulse generation, memory section and filtering and detection. Both signal and control pulses originate from a CW laser at the control-field frequency. EOM 1 first carves the CW light into pulses. A 90:10 beam splitter (BS) then splits the laser into a high-power control path (90%) and a low-power signal path (10%). The laser in the signal path is later shifted to the signal frequency via EOM 2. The two beams are recombined via a BD before entering the memory cell for storage and retrieval. Post-retrieval filtering comprises a GTP, three double-passed etalons and a bandpass filter (BP). The signal is finally routed to SNSPDs for detection. Using multiple detectors helps mitigate detector dead time effects and enables higher overall count rates. DM, dichroic mirror; HWP, half-wave plate; PBS, polarizing beam splitter; PC, Pockels cell; M, mirror; WPs, a half-wave plate and a quarter-wave plate. **b**, Pulse sequences. The diagram illustrates the timing of the signal and control pulses, along with the gating windows of the optical pumping and the Pockels cell.

Further improvements in precision require reducing mode crosstalk (as indicated in equation (4)), which primarily arises from the storage process. A key limitation is the trade-off between crosstalk and storage efficiency, as higher efficiency leads to increased crosstalk (see Supplementary Section 6 for discussions and simulations). Recent advances such as the efficiency enhancement via light–matter interference (EEVI) protocol^51^ offer a route to high-efficiency storage while preserving mode selectivity. Cavity-enhanced Raman memories offer a similar solution^52^, albeit with bandwidth constraints set by cavity finesse. Alternatively, optimal control techniques can tailor pulse shapes to simultaneously maximize storage efficiency and suppress crosstalk^53,54^. Moreover, the practical advantage of super-resolving measurements, quantified by FI per input photon, is currently limited by system photon loss. Similar to other TF super-resolution platforms, our system exhibits a low end-to-end efficiency of about 0.3%, despite an internal efficiency of 14.6% ± 0.1%. This drop is primarily due to stringent filtering required to suppress the copropagating control field, which is detuned by just 9.2 GHz. Achieving the necessary 4 × 10^7^ extinction ratio requires a series of lossy optics (Fig. 5). By contrast, ladder-type quantum memories, such as fast-ladder memory^55^ and off-resonant cascaded absorption memory (ORCA)^56,57^, exploit a higher-lying excited state alongside widely separated, counter-propagating signal and control fields. This configuration enables efficient control suppression with minimal loss. These systems have demonstrated noise levels as low as 10^−5^ photons per signal photon^56^ and end-to-end efficiencies up to 35% (ref. ^55^). Integrating our high-fidelity mode-selective measurement scheme with such architectures could enable unconditional TF super-resolution.

## Methods

### Experimental set-up

Our Raman memory set-up is shown in Fig. 5a. The storage medium consists of a 75-mm-long caesium (Cs-133) vapour cell with a three-layer μ-metal magnetic shielding. We heated the cell to 105 ± 1 ^∘^C using a quad-twisted cryogenic wire, achieving an effective optical depth of 4.8 ± 0.1 × 10^3^. The atoms were initialized to the initial state $| g\rangle$ by an external cavity diode laser (ECDL, Toptica DL100) driving the D1 transition (6^2^**S**_1/2_ → 6^2^**P**_1/2_). EOM 3 (EOSpace 850UL) gated the pump laser, switching it off during the experimental window. We achieved a pumping efficiency of 99.6% ± 0.1%.

We operated our memory with a detuning Δ = 2Δ_*hf*_ to suppress four-wave-mixing noise^58^. Both the signal and control fields were derived from a single continuous wave (CW) ECDL laser (Toptica DL Pro) operating at the control frequency (351.7122 THz). An arbitrary waveform generator (AWG, Tektronix AWG70001A) drove the EOM 1 (Sacher Lasertechnik AM830PF) to carve the CW laser into pulses. The laser was then split using a 90:10 beam splitter, directing 90% of the optical power to the control field path. The remaining 10% went to EOM 2 (New Focus 4851), which generated a sideband at the signal frequency (351.7030 THz). We selected this sideband as the signal field by using a Fabry–Pérot etalon with a free spectral range (FSR) of 36.8 GHz. The signal field was delayed by 437 ns using a long fibre to achieve temporal synchronization with the control pulses, ensuring their temporal overlap for memory storage and retrieval. Before entering the memory, the signal field also passed through a Pockels cell to remove residual CW background in the retrieval window. The polarization of the signal and control fields was set to orthogonal for memory interaction, allowing their combination using a BD before being focused into the vapour cell.

We used a pair of convex lenses to focus the signal, control and pumping beams into the vapour cell to enhance the interaction strength. The beam widths at the focus were approximately 164 ± 5 μm for the signal and 190 ± 5 μm for the control. As shown in Fig. 5b, the first signal pulse temporally overlaps with the control pulse Ctrl_stor_ for storage. After a storage time of 200 ns, the control pulse Ctrl_retr_ retrieves the stored signal. The third signal pulse serves as a reference for the signal pulse power. All signal and control pulses share the same Gaussian envelope width parameter as a fundamental Gaussian pulse (HG_0_) with an electric field full width at half maximum of 50 ns.

After the memory, we suppressed the control and pump fields using a GTP, followed by three double-passed etalons (two with an FSR of 18.4 GHz and one with an FSR of 103 GHz) and a bandpass filter (central wavelength 850 nm, full width at half maximum bandwidth 10 nm). Finally, we split the signal into four equal parts and detected them using four SNSPDs (Photon Spot). Using multiple detectors helps mitigate detector dead time effects and enables higher overall count rates. The detected counts were registered using a time tagger (Swabian TimeTagger20).

### Signal preparation

The signal in our separation estimation task is an incoherent mixture of two Gaussian spectral lines. Its frequency-domain representation can be written as

9 $$\varPsi (\omega )=\frac{1}{\sqrt{2}}\left[\exp \left(-i\frac{\phi }{2}\right)\psi \left(\omega -{\omega }_{0}-\frac{\epsilon \sigma }{2}\right)+\exp \left(i\frac{\phi }{2}\right)\psi \left(\omega -{\omega }_{0}+\frac{\epsilon \sigma }{2}\right)\right],$$

where $\phi \in (-{{\uppi }},{{\uppi }}]$ is a random phase between the two spectral lines. Here, we carved the temporal profile of our signal field as the inverse Fourier transform of equation (9),

10 $$\varPsi (t)=A\cos \left(\frac{\epsilon \sigma t-\phi }{2}\right)\exp \left(-{t}^{2}{\sigma }^{2}\right),$$

where *A* is the amplitude. The signal pulse has a spectral width of *σ* = 33.3 Mrad s^−1^ (5.30 MHz). In our experiment, we generated signals with separation parameters *ϵ* from 0 to 1 with an increment of 0.05 (0.265 MHz). To introduce the incoherence of the two spectral lines, we prepared the signal in a mixture of four different relative phases, *ϕ* = −π/2, 0, π/2, π, for each separation.

One key requirement for this experiment is the accurate generation of signal and control pulses. To ensure high-fidelity pulse carving, we characterized the frequency response of our electronic pulse generation system, which comprises the AWG, a radiofrequency (RF) splitter and two RF amplifiers that drive the EOM 1. By measuring the system’s frequency response function, we applied a correction to the input signals of the AWG, compensating for frequency-dependent variations in the RF components. This correction ensures uniform amplification across all frequency components, minimizing distortions in the carved pulses. The measured intensity pulse shapes of HG_0_ and HG_1_ are presented in Supplementary Section 7.

### Data collection and analysis

For each signal, comprising one separation and one phase setting, we first stored it using a control pulse of HG_0_ with a pulse energy of 130 ± 1 pJ. At this control energy, our memory typically operates with a storage efficiency of 39.5% ± 0.6% and a total internal efficiency of 14.6% ± 0.1% for a storage duration of 200 ns. The stored signal was retrieved using a control pulse in the HG_1_ mode, with the same pulse energy as the HG_0_ write-in pulse. The HG_1_ mode was chosen to minimize distortion in the pulse sequence owing to its symmetrical temporal profile. Importantly, the choice of retrieval mode has a negligible impact on the retrieval efficiency. The pulse sequence was repeated every 3 μs, with a total measurement time of 2 s. To accurately account for signal background noise and dark counts, we also recorded data with the control pulse blocked for an equivalent duration. The counts detected in the retrieval time window during this control-blocked run were later used to subtract the noise counts from the retrieved signal counts to minimize the estimator bias caused by the noise. The above procedures were repeated for all four phases. Subsequently, we modified the control read-in pulse to the HG_1_ mode while keeping the read-out pulse unchanged and repeated the measurements. Finally, the entire experiment was repeated for all separations of interest.

To obtain the final detected counts, we first identified a reference pulse count that yielded approximately *N* = 2 × 10^3^, 10 × 10^3^ or 100 × 10^3^ total retrieval counts (with a variation of less than 5%) of the signals across all combinations of the two control read-in pulses and four phases. For each separation, we used the reference counts to randomly sample a subset that yielded *N* retrieved counts from the data files. The corresponding noise counts were subtracted from the retrieved counts to correct the background contributions. This random sampling approach, combined with the use of reference counts, was used to mitigate the impact of signal power fluctuations on the final estimation process. The retrieval counts acquired for HG_0_ and HG_1_ storage, *N*_0_ and *N*_1_, respectively, were used directly to compute the raw estimator. We repeated the entire sampling and analysis procedure 50 times using bootstrapping to estimate the variances and MSEs of the estimators. To obtain the error bars for the MSE shown in Fig. 3, we repeated the bootstrapping process ten times to get ten datasets and calculated the standard deviations of the MSE.

### System calibration

To characterize the crosstalk matrix *M* of the experimental system and the perturbed HG projection probabilities $\widetilde{P}(0| \epsilon )$ and $\widetilde{P}(1| \epsilon )$ in equation (5), we used a dataset for separations *ϵ* from 0 to 1 with *N* = 1.6 × 10^5^ detected photon counts for each separation. For each separation, we normalized the retrieved counts *N*_0_ and *N*_1_ corresponding to the two HG projections to obtain the relative frequencies *f*_0_ = *N*_0_/(*N*_0_ + *N*_1_) and *f*_1_ = *N*_1_/(*N*_0_ + *N*_1_). The values of *α* and *β* were estimated by minimizing the least-squares cost

11 $$C(\,{f}_{0},{f}_{1}| \alpha ,\beta )=\mathop{\sum }\limits_{\epsilon }\{{[\,{f}_{0}(\epsilon )-\widetilde{P}(0| \epsilon )]}^{2}+{[\,{f}_{1}(\epsilon )-\widetilde{P}(1| \epsilon )]}^{2}\}.$$

The perturbed probabilities, parameterized by *α* and *β*, were compared with the measured relative frequencies, and the sum of squared residuals across all separations was computed. We then optimized the parameters using nonlinear least-squares minimization. The resulting values characterize the performance of our experimental system and were subsequently used to compute the MLE estimates.

### Reporting summary

Further information on research design is available in the Nature Portfolio Reporting Summary linked to this article.

## Supplementary information

Supplementary Information Supplementary Sections 1–7 and Figs. 1–6.

Reporting Summary

Peer Review File

## Data Availability

The data that support the findings of this study are included within this article and its Supplementary Information. The processed photon count data files are available via Zenodo at 10.5281/zenodo.19351042 (ref. ^59^).

## Code availability

The code used for data acquisition and data analysis is available via Zenodo at 10.5281/zenodo.19351042 (ref. ^59^).
