# Supplementary material for: Super-resolving frequency measurement with mode-selective quantum memory
Source: Nat Sens. 2026 May 15;1(7):627–35. doi: 10.1038/s44460-026-00073-9 (PMC13368581; doi:10.1038/s44460-026-00073-9)
Supplement: Supplementary file 1 — Supplementary Sections 1–7 and Figs. 1–6. [file 44460_2026_73_MOESM1_ESM.pdf]

nature sensors

Supplementary information https://doi.org/10.1038/s44460-026-00073-9

Super-resolving frequency measurement with mode-selective quantum memory

In the format provided by the authors and unedited

Contents

1 Fundamental bias at small separations **2**

2 Quantum memory interaction **4**

3 Different retrieval times and retrieval modes **5**

4 Comparison of time–frequency super-resolution sensor architectures **6**

5 Sensing applications in Doppler LiDAR **6**
5.1 Single target velocity measurement 7
5.2 Joint estimation of range and velocity 8
5.3 Resolving two or more objects 8

6 Sources of mode crosstalk **8**

7 Precise pulse carving **9**

1 Fundamental bias at small separations

In the maximum likelihood estimation (MLE) process, the non-negativity constraint of the parameter space Θ introduces a positive bias at very small frequency separations, where the distribution of an ideally unbiased estimator may extend into the negative region. Here, we present both an analytical analysis and numerical simulations to characterize this fundamental bias of a two-mode demultiplexing system with crosstalk matrix $\boldsymbol{M}$ (Eq. 3 in the main text).

In the absence of crosstalk, the ideal detection probability for mode n is

$$P(n|\epsilon) = \frac{\epsilon^{2n}}{16^n n!} \exp\left(-\frac{\epsilon^2}{16}\right). \tag{S1}$$

And the Fisher information for this Hermite-Gaussian (HG) measurement basis can be calculated as

$$\mathcal{F}_{\mathrm{HG}}(\epsilon) = \sum_{n=0}^{\infty} \frac{1}{P(n|\epsilon)} \left(\frac{\partial P(n|\epsilon)}{\partial \epsilon}\right)^2. \tag{S2}$$

Considering only the first two modes, the normalized probability of projecting onto the HG_1 mode after perturbation by the crosstalk matrix $\boldsymbol{M}$ is

$$\tilde{P}(1|\epsilon) = \beta - \frac{16(\alpha+\beta-1)}{16+\epsilon^2}, \tag{S3}$$

and the HG_0 projection probability is $\tilde{P}(0|\epsilon) = 1 - \tilde{P}(1|\epsilon)$. The exact expression for the Fisher information of the non-ideal two-mode projection method can be derived from Eq. S2 in the main text as follows:

$$\mathcal{F}(\epsilon) = \frac{(\alpha+\beta-1)^2(\epsilon/4)^2}{4(1+(\epsilon/4)^2)^2(\alpha+(1-\beta)(\epsilon/4)^2)(1-\alpha+\beta(\epsilon/4)^2)}. \tag{S4}$$

We assume the mode crosstalk between the two modes is low, i.e. $\alpha \approx 1$ and $\beta \approx 1$, and the separation is small. Then Eq. S4 can be approximated as

$$\mathcal{F}(\epsilon) \approx \frac{1}{4\left[(1-\alpha)/(\epsilon/4)^2 + 1\right]}, \tag{S5}$$

which is the Eq. 4 in the main text. Using maximum likelihood estimation (Eq. 5 in the main text), the likelihood function is given by

$$\mathcal{L} = N_0 \ln \tilde{P}(0|\epsilon) + N_1 \ln \tilde{P}(1|\epsilon), \tag{S6}$$

where N_0 and N_1 represent the experimentally measured detection counts for HG_0 and HG_1 projections, respectively, with the total count given by $N = N_0 + N_1$. The analytical solution of the MLE estimator can be obtained by solving the critical point equation $d\mathcal{L}/d\epsilon = 0$, yielding $\hat{\epsilon} = \sqrt{\frac{16(N_1-(1-\alpha)N)}{\beta N - N_1}}$. However, this solution is valid only when both the numerator and denominator are non-negative; thus, we require $N_1 \geq (1-\alpha)N$ (We neglect the condition $N_1 < \beta N$, as the probability of $N_1 > \beta N$ is negligible for small separations and low mode crosstalk). This condition corresponds to the range of the modal prediction discussed in the main paper. If a combination of N_0 and N_1 values falls outside this range, the likelihood function $\mathcal{L}$ achieves its maximum at the non-differentiable boundary point $\epsilon = 0$. In such cases, the MLE defaults to $\hat{\epsilon} = 0$. Therefore, the MLE estimators are

$$\hat{\epsilon}_{\mathrm{MLE}} = \begin{cases} \sqrt{\frac{16(N_1-(1-\alpha)N)}{\beta N - N_1}}, & \text{if } N_1 \geq (1-\alpha)N, \\ 0, & \text{if } N_1 < (1-\alpha)N. \end{cases} \tag{S7}$$

The maximum likelihood estimation method inherently produces only non-negative estimators based on the detected counts. When N_0 and N_1 result in a negative estimate, the estimator is set to zero. Consequently, this introduces a bias toward positive values, as all negative estimates are truncated to zero.

Since we employ photon count detection, the detection counts N_0 and N_1 follow Poisson distributions. Specifically, we model N_1 as Poisson(μ_1), where $\mu_1 = \tilde{P}(1|\epsilon)N$. Substituting this distribution into the estimator

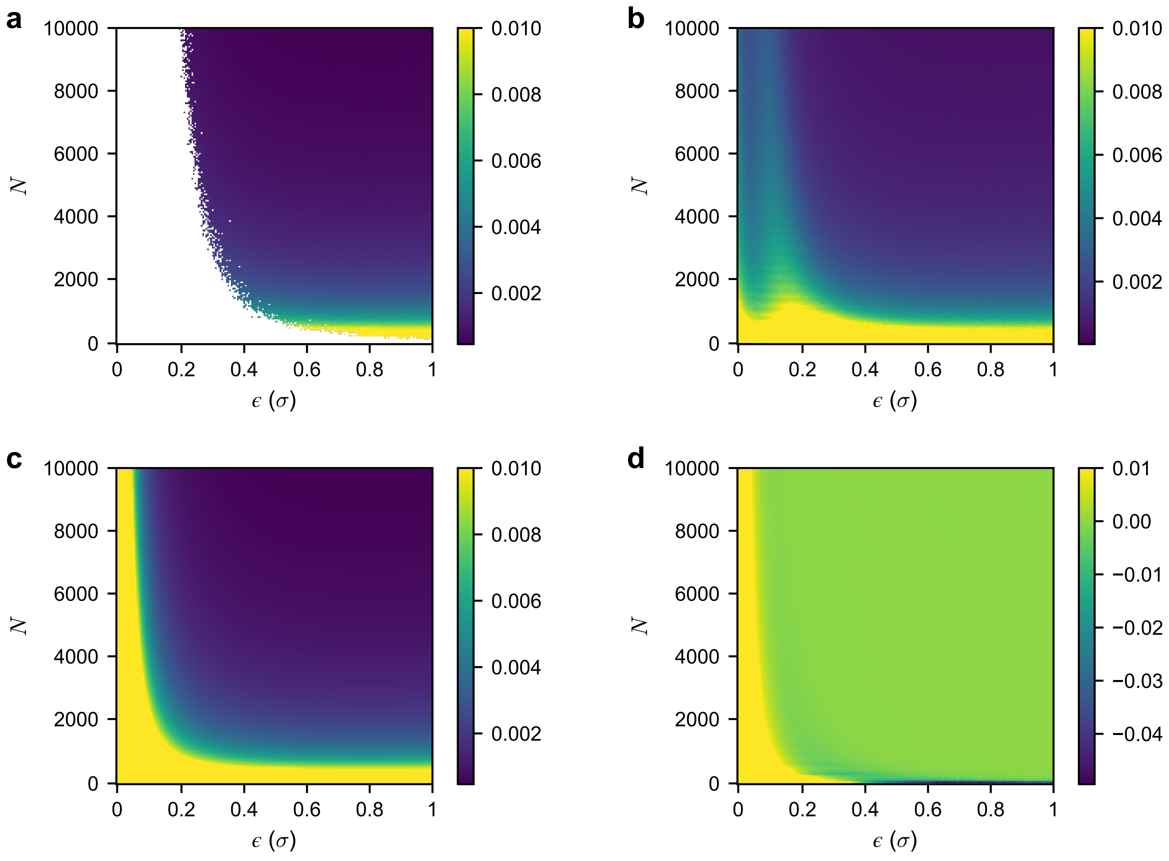

Fig. S1 Simulation results of the mean square error and the unbiased Cramér-Rao lower bound for maximum likelihood estimation. (**a**) Simulated MSE, where the white region indicates the onset of truncation due to the non-negativity constraint. (**b**) MSE with the non-negativity constraint applied. (**c**) The unbiased CRLB. (**d**) The difference between the CRLB and the MSE (CRLB − MSE).

formula in Eq. S7, we derive the mean square error (MSE) as

$$\mathrm{MSE}(\epsilon, N) = \sum_{k=0}^{(1-\alpha)N-1} \epsilon^2 \cdot \frac{\mu_1^k e^{-\mu_1}}{k!} + \sum_{k=(1-\alpha)N}^{\beta N-1} \left(4\sqrt{\frac{k-(1-\alpha)N}{\beta N - k}} - \epsilon\right)^2 \cdot \frac{\mu_1^k e^{-\mu_1}}{k!}. \tag{S8}$$

(Here, we neglected the $N_1 > \beta N$ part, as it is extremely unlikely to occur for small separations when $\beta \approx 1$.)

The MSE simulated using our experimentally measured crosstalk is shown in Fig. S1 as a function of the separation ϵ and the number of photons N. In panel (a), the white region indicates where the MLE estimators begin to truncate negative estimates, and panel (b) displays the corresponding MSE with this truncation applied. Panel (c) presents the unbiased Cramér-Rao lower bound (CRLB). By taking the difference between the unbiased CRLB and the MSE (CRLB − MSE), we obtain panel (d), which highlights a transition: for large separations, the MSE exceeds the unbiased CRLB (blue regions), but as the separation decreases and with larger bias, it drops below the CRLB (yellow regions).

2 Quantum memory interaction

To characterize the mappings between the signal mode and the spin-wave mode, we can describe the mapping using Green's functions [1], as the Raman memory equations are linear:

$$B_{\text{stor}}(z) = \int_{-\infty}^{+\infty} \mathrm{d}\tau\, K_1(z,\tau) S_{\text{in}}(\tau), \tag{S9}$$

$$S_{\text{out}}(\tau) = \int_{0}^{L} \mathrm{d}z\, K_2(z,\tau) B_{\text{stor}}(z), \tag{S10}$$

where $S_{\text{in}}(\tau)$ is the input signal mode, $B_{\text{stor}}(z)$ is the stored spin-wave mode, $S_{\text{out}}(\tau)$ is the retrieved optical mode. $K_1(z,\tau)$ and $K_2(z,\tau)$ are the corresponding Green's function kernels that characterize the storage and retrieval processes and contain information about the corresponding control fields, such as temporal modes and intensities.

To derive the memory efficiencies, we first need to define the number of excitations N in each process.

$$N_{\text{in(out)}} = \int_{-\infty}^{\infty} d\tau \left\langle S^{\dagger}_{\text{in(out)}}(\tau) S_{\text{in(out)}}(\tau) \right\rangle \tag{S11}$$

$$N_{\text{stor}} = \int_{0}^{L} dz \left\langle B^{\dagger}_{\text{stor}}(z) B_{\text{stor}}(z) \right\rangle . \tag{S12}$$

where $N_{\text{in(out)}}$ is the average number of input (output) excitations in the optical field, and N_{stor} is the average number of the stored spin-wave excitations in the medium. Thus, the storage, retrieval and total memory efficiencies are given as

$$\begin{aligned} \eta_{\text{storage}} &= N_{\text{stor}}/N_{\text{in}} = 1 - N_{\text{tran}}/N_{\text{in}} \\ \eta_{\text{retrieval}} &= N_{\text{out}}/N_{\text{stor}} = N_{\text{out}}/(N_{\text{in}} - N_{\text{tran}}) \\ \eta_{\text{total}} &= N_{\text{out}}/N_{\text{in}}. \end{aligned} \tag{S13}$$

where $N_{\text{tran}} = N_{\text{in}} - N_{\text{stor}}$ is the transmitted number of excitations. Experimentally, we used the transmitted photon counts to calculate the storage efficiency, as the number of excitations is not accessible.

The modal properties of the Raman memory can be understood by performing singular value decomposition on the Green functions $K_1(z,\tau)$ and $K_2(z,\tau)$ [1]. For the storage process, $K_1(z,\tau)$ can be decomposed into

$$K_1(z,\tau) = \sum_k \lambda_k \psi_k(z) \phi^*_k(\tau) \tag{S14}$$

where λ_k are the eigenvalues of the decomposition, sets of $\psi_k(z)$ and $\phi_k(\tau)$ represent the orthonormal bases of spin-waves and input temporal modes, respectively. In a single-mode quantum memory, only a specific input temporal mode $\phi_1(\tau)$ is efficiently coupled and stored as a corresponding spin-wave mode $\psi_1(z)$, while all orthogonal modes $\phi_k(\tau)$ for $k \neq 1$ pass through the medium unaffected. This selective interaction effectively allows the memory to function as a mode filter, storing one targeted signal while leaving others undisturbed. Crucially, the mode that is stored can be tuned by shaping the control pulse used in the Raman interaction. Adjusting the pulse's shape, timing, and intensity modifies the Green function $K_1(z,\tau)$, which in turn determines the set of temporal modes $\phi_k(\tau)$ and their associated coupling strengths, represented by eigenvalues λ_k.

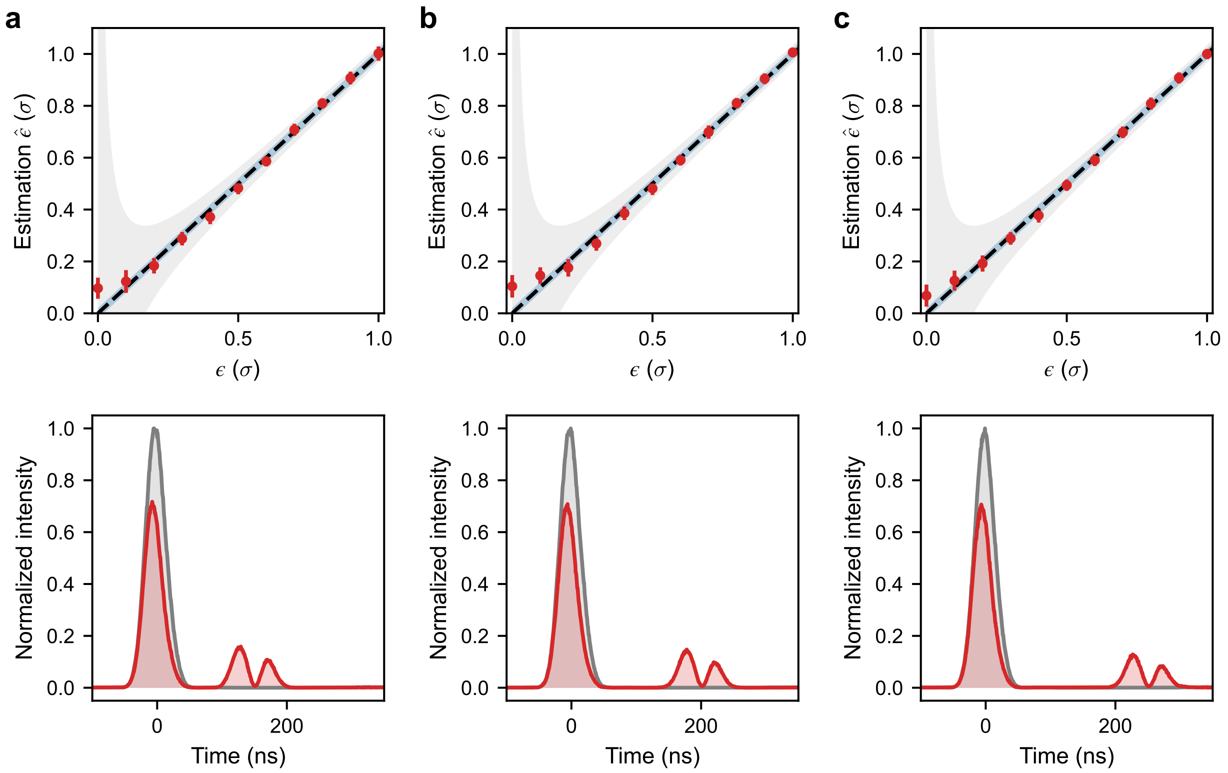

Fig. S2 Performance of maximum likelihood estimation at different retrieval times. (**a**), (**b**), and (**c**) correspond to retrieval times of 150 ns, 200 ns, and 250 ns, respectively. In each case, the upper panels show the MLE estimates (red dots), along with the quantum bounds (blue region) and the direct intensity measurement bounds (gray region). The total detection count is 10^4. Red dots and error bars denote the mean and standard deviation derived from 50 bootstrap resamples. The lower panels display the detected signal pulse sequences for two spectral lines with zero separation and zero relative phase. The input signal is shown in gray; the first red pulse indicates leaked signal, and the second red pulse corresponds to the retrieved signal. The read-in control pulse is in the HG_0 mode, and the read-out control pulse is in the HG_1 mode.

3 Different retrieval times and retrieval modes

Photonic quantum memories are designed to store photons over user-defined delay times. Raman quantum memories, in particular, offer additional functionality such as temporal mode conversion, where the stored signal can be retrieved into a temporal mode defined by the read-out control pulse. These combined capabilities of storage and mode conversion make Raman memories more versatile than previously demonstrated mode-filtering super-resolution schemes, and could enable applications like distributed sensing networks, as discussed in the main text. Here, we demonstrate the super-resolution performance of a Raman memory using different retrieval times and output temporal modes.

The maximum storage time is generally limited by thermal diffusion in the warm vapor cell: atoms carrying the spin-wave coherence drift out of the interaction region (defined by the control beam size), rendering the stored signal unrecoverable. The typical coherence time is on the order of microseconds [2]. In our experiments, we characterize the super-resolution performance of the Raman memory at storage times of 150 ns, 200 ns, and 250 ns, as shown in Fig. S2. These durations are chosen based on constraints from our delay fiber length and pulse sequence design. For all three retrieval times, the super-resolution performance remains comparable, as illustrated in the upper panels of the figure. The lower panels show the raw detection counts from a representative experimental run, where the input signal had zero frequency separation and zero relative phase. The retrieved signals appear as the second red pulse, occurring precisely at 150 ns, 200 ns, and 250 ns, corresponding to retrieval using an HG_1 mode as the control read-out pulse.

We also performed the experiment using different retrieval modes to demonstrate the flexibility in choosing the read-out control pulse. The results are shown in Fig. S3, where the control read-out pulses are set to HG_0 and HG_1, respectively. The super-resolution performance remains comparable in both cases, and the retrieved signals (the second red pulse) clearly exhibit the corresponding temporal mode shapes.

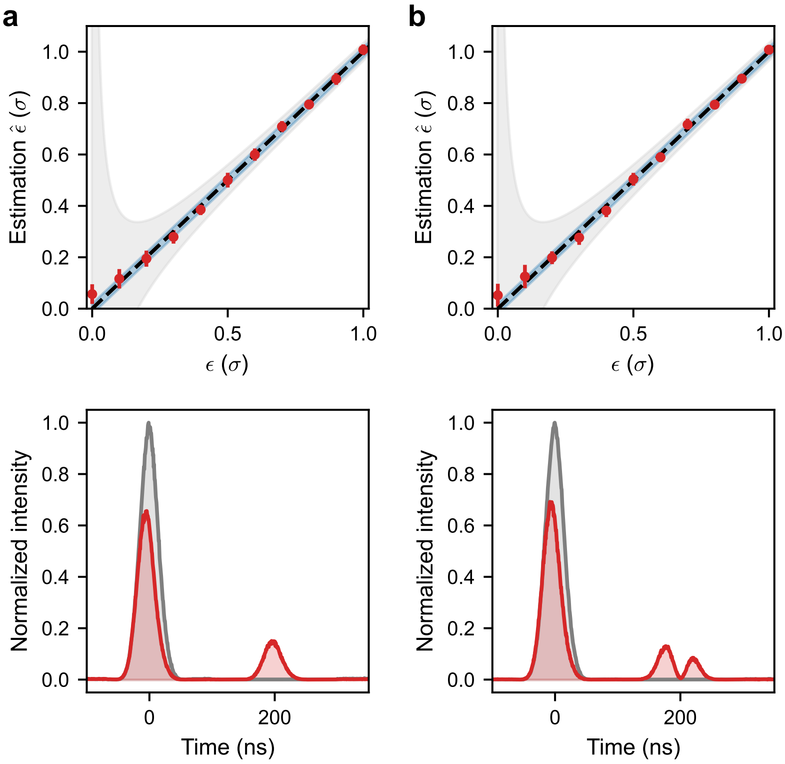

Fig. S3 Performance of maximum likelihood estimation with different retrieval modes. (**a**) and (**b**) correspond to retrieval control modes of HG_0 and HG_1, respectively. The upper panels show the MLE estimates (red dots), along with the quantum bounds (blue region) and the direct intensity measurement bounds (gray region). The total detection count is 10^4. Red dots and error bars denote the mean and standard deviation derived from 50 bootstrap resamples. The lower panels display the detected signal pulse sequences for two spectral lines with zero separation and zero relative phase. The input signal is shown in gray; the first red pulse indicates leaked signal, and the second red pulse corresponds to the retrieved signal. The read-in control pulse is in the HG_0 mode.

4 Comparison of time–frequency super-resolution sensor architectures

Table S1 presents a detailed comparison of frequency-domain super-resolution schemes [3–5], evaluating their underlying principles, programmability, complexity, bandwidth, and precision enhancement. While PuDTAI (GEM) and Raman protocols both utilize quantum memories, the GEM architecture relies on mapping frequency components to longitudinal spatial positions via magnetic field gradients. This mechanism inherently restricts the system to the ultra-narrowband regime, while necessitating cumbersome magnetic field gradients and laser-cooling infrastructure. Pushing to larger bandwidth requires a steeper gradient to span a wider detuning range across a fixed-length medium. Because the total optical depth is finite, this effectively reduces the optical depth per unit frequency, causing storage efficiency to drop as bandwidth increases. At the same time, the steeper gradient compresses the spatial mapping, shrinking the spatial separation between frequency components and making the stored coherence more susceptible to blurring from atomic motion. These physical constraints are compounded by engineering limits, since steeper gradients demand higher currents and more coil turns, increasing inductance and complicating the rapid gradient reversal required for retrieval. Furthermore, PuDTAI and SUSI are restricted to a fixed basis of symmetric and antisymmetric modes. Conversely, while QPG offers high programmability, it lacks storage capabilities and operates in a very high bandwidth regimes ($>$ 100 GHz).

5 Sensing applications in Doppler LiDAR

The mode-selective capability of our Raman quantum memory platform offers a distinct advantage for remote sensing applications, particularly in Doppler Light Detection and Ranging (LiDAR). In this section, we outline the operational principles of Doppler LiDAR and discuss how our platform enables high-precision velocity and range estimation.

Table S1 Comparison of time–frequency super-resolution sensor architectures

Scheme	QPG	PuDTAI	SUSI	Raman
**Principle**	Sum-frequency generation with shaped pulses	Time-inversion interferometry with spatial-spectral mapping	Spectral-domain inversion interferometry	Stimulated Raman scattering with pulse shaping
**Programmability**	Arbitrary mode	Fixed (symmetric/ antisymmetric)	Fixed (symmetric/ antisymmetric)	Arbitrary mode
**Storage & Conversion**	No	Storage	No	Yes
**Complexity**	Low (nonlinear waveguide, spatial-light modulator pulse shaping)	High (cold atoms, magnetic gradients, AC-Stark modulation)	Medium (interferometer stabilization, electro-optic lenses)	Low (warm vapor, electro-optical modulator pulse shaping)
**Bandwidth**	100s of GHz to THz	kHz to low MHz	10s of GHz to 100s of GHz	100s of kHz to low GHz
**Precision enhancement**	~ 24	~ 20	~ 2.13	~ 37

Doppler LiDAR systems generally fall into two categories: coherent [6] and incoherent [7] detection. Coherent Doppler LiDAR relies on heterodyne detection, where the backscattered signal is mixed with a local oscillator (LO) to extract the Doppler frequency shift. While offering shot-noise-limited sensitivity, coherent Doppler LiDAR requires a narrow-linewidth laser, spatial mode matching between the signal and a phase-locked LO, and is susceptible to performance degradation from atmospheric turbulence and speckle noise. On the other hand, incoherent Doppler LiDAR typically utilizes passive optical filters (such as Fabry-Pérot etalons or molecular absorption cells) with steep transmission slopes. The Doppler frequency shift is converted into an intensity variation as the signal moves along the filter's transmission edge. However, its performance and precision is fundamentally limited by the steepness of the optical filters and the trade-off between dynamic range and sensitivity.

Our Raman memory-based platform provides a high-precision, mode-selective measurement in an incoherent way that overcomes the above limitations. By performing mode-selective storage, our method offers a distinct advantage over static edge filters: the spectral properties of the filter are directly defined by the optical control field, allowing the measurement basis (center frequency and bandwidth) to be flexible and dynamically programmable. This mode-selectivity also enhances noise resilience. Unlike conventional incoherent LiDARs that integrate background noise throughout the filter window, our system functions as a temporal filter, effectively rejecting any noise components that are temporally or spectrally orthogonal to the control mode. Moreover, unlike coherent LiDAR, our approach does not require a phase-stable local oscillator, making it resilient to noise from turbulence and phase decorrelation.

By leveraging single-photon detection and optimal mode filtering, our platform achieves fine resolution in low signal-to-noise ratio (SNR) regimes, where conventional methods fail. A compelling application is remote vibrometry, applicable to scenarios such as laser ultrasonic testing for defect detection in industrial composites and non-contact photoacoustic sensing in biomedical imaging, where a stationary target exhibits surface oscillations in the MHz regime. For instance, a target vibrating with multiple acoustic frequencies will generate a return signal composed of closely spaced spectral lines. enabling high-sensitivity at MHz-GHz high speed acoustic monitoring even in the presence of turbulence. In the following sections, we detail specific scenarios where this technology can be applied as a Doppler LiDAR.

5.1 Single target velocity measurement

Consider a scenario where a pulsed laser with spectral width σ is emitted and reflected by a single moving target, acquiring a Doppler frequency shift δ proportional to its velocity. To measure this frequency shift, we employ a mode-selective measurement that directly infers the frequency shift from the detected photon counts. Through a pre-calibration of the storage efficiency η as a function of signal central frequency, the unknown shift δ is retrieved by comparing the measured photon statistics of the filtered signal. Crucially, this platform fills a technological gap in the MHz to GHz bandwidth regime, a domain where conventional direct intensity techniques (such as grating or Fourier transform spectrometers) are inaccessible. Furthermore, unlike passive filters, the spectral position and bandwidth of our acceptance mode can be dynamically adjusted, effectively allowing us to select the optimal "edge" to maximize sensitivity for the expected velocity range.

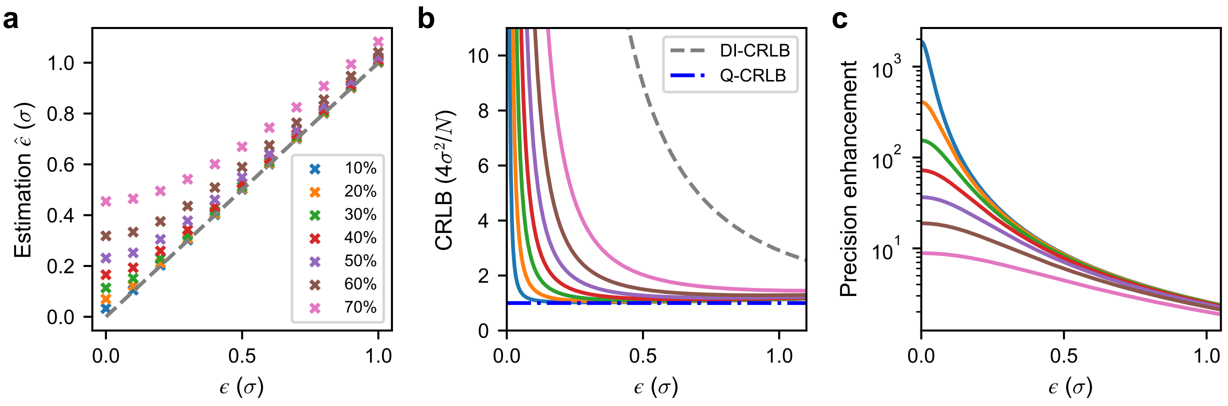

Fig. S4 Effect of storage efficiency on super-resolution performance. (**a**) Raw estimators $\hat{\epsilon} = 4\sqrt{N_1/N_0}$ for various storage efficiencies, showing increased bias at higher efficiencies due to stronger temporal mode crosstalk. (**b**) Unbiased CRLB for the two-mode demultiplexing method at different storage efficiencies, compared to the quantum CRLB and the CRLB for the direct intensity measurement method. (**c**) Precision enhancement $\mathcal{F}/\mathcal{F}_{\mathrm{DI}}$ as a function of separation ϵ, demonstrating logarithmic improvement for small separations.

5.2 Joint estimation of range and velocity

Conventional Doppler LiDAR systems relying on very narrowband lasers often provide poor resolution in range estimation due to the fundamental Fourier limit. By operating with high-bandwidth pulses, our platform is capable of simultaneously estimating range and velocity with high resolution. In this scenario, the return signal is characterized by both a time delay τ (encoding range) and a frequency shift δ (encoding velocity). Since the Raman interaction is strictly mediated by the control field, the storage process effectively projects the input signal onto a specific time-frequency mode basis defined by the control pulse's temporal envelope and central frequency. This allows for the optimization of the measurement to maximize Fisher Information for both parameters.

5.3 Resolving two or more objects

A more complicated scenario involves two or more targets moving at slightly different velocities [8]. The return signal becomes an incoherent mixture of two or more time-frequency modes: $\rho = \sum_i p_i |\psi(t-\tau_i, \omega - \delta_i)\rangle\langle\psi(t-\tau_i, \omega-\delta_i)|$. This scenario maps directly to resolving closely spaced spectral lines addressed in our work. When the velocity difference is sub-Rayleigh and the two spectral lines overlap, conventional systems struggle to resolve the two velocities. By shaping the control field, we can perform an optimized mode-selective measurement for resolving range and velocity of two or more objects. In many practical LiDAR and vibrometry scenarios, decoherence occurs when reflecting off rough surfaces (diffuse scattering) or propagating through atmospheric turbulence, where the relative phases between reflections from different scattering centers are randomized. Consequently, the interference terms average to zero, and the return signal is accurately described by the incoherent mixed state ρ. Our method is therefore particularly advantageous for sensing diffuse targets, where traditional coherent detection (heterodyne) is often degraded by speckle noise and wavefront distortions.

6 Sources of mode crosstalk

In this work, we employed an imperfect two-mode projection method to generate our estimators, incorporating all imperfections into the parameters α and β in the perturbation matrix $\boldsymbol{M}$ (Eq. 3 in the main text). Below, we discuss the primary sources of mode crosstalk and the resulting bias in the raw estimator.

The primary source of temporal mode crosstalk arises from the memory's storage interaction. Although the memory operates approximately in a single-mode regime, increasing the control field strength induces stronger AC Stark shifts. This leads to the coupling of orthogonal signal modes into the spin wave, distorting the ideal projection probabilities and introducing bias. Fig. S4 illustrates how different storage efficiencies (i.e. different control read-in pulse power) affect the super-resolution performance and estimation precision relative to direct intensity (DI) measurement methods.

In Fig. S4, panel (a) shows the raw estimators $\hat{\epsilon} = 4\sqrt{N_1/N_0}$ for different storage efficiencies. As storage efficiency increases, so does crosstalk, resulting in greater bias in the raw estimators. In panel (b), we apply

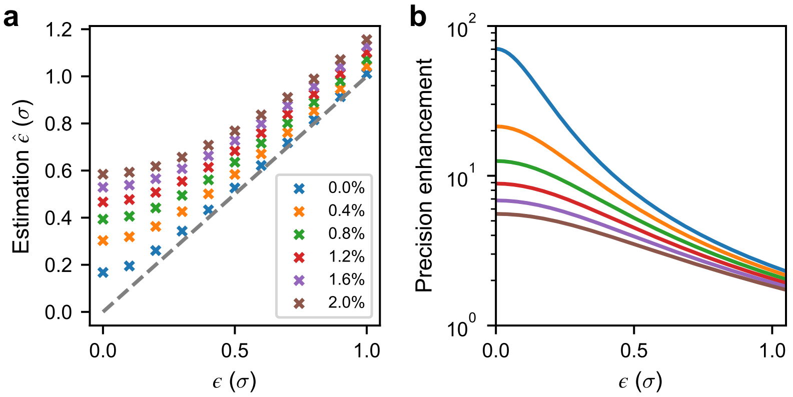

Fig. S5 Effect of control leak counts on estimation bias and precision. (**a**) The bias in the raw estimators increases with control leakage, expressed as a percentage of the total detection counts. (**b**) Precision enhancement of the mode filtering method decreases as background noise increases. Storage efficiency is fixed at 40%.

maximum likelihood estimation and plot the corresponding unbiased CRLB for the two-mode demultiplexing method, alongside the quantum CRLB and the CRLB for DI methods. Higher storage efficiency leads to an increase in the CRLB, resulting in reduced estimation precision. This trend is further quantified in panel (c), where the precision improvement factor, $\mathcal{F}/\mathcal{F}_{\mathrm{DI}}$, is plotted as a function of separation ϵ. As the storage efficiency decreases, the super-resolution parameter increases nearly logarithmically. However, very high precision improvement using very low storage efficiencies is impractical in experiments, as fewer signal photons are retrieved, constant background and control field leakage reduce the signal-to-noise ratio, potentially increasing the observed crosstalk and estimation bias.

Another major limiting factor contributing to excess crosstalk in our experiment is control field leakage, which introduces a constant background noise. Due to the small frequency separation (9.2 GHz) between the signal and control fields, completely filtering out the control field is technically challenging. As a result, residual control photons are detected and contribute equally to both HG_0 and HG_1 projection measurement counts, adding uniform noise across all separations that distorts the projection probabilities.

To quantify this effect, we simulate how constant background counts influence the bias of the raw estimators and the precision improvement of MLE estimators. In this simulation, we fix the storage efficiency at 40% and introduce a constant leakage level ranging from 0 to 2% of the total detected counts. The results are shown in Fig. S5. Panel (a) shows that increasing control leakage leads to larger bias in the raw estimator, similar to the effect of increasing storage efficiency. The rate of bias increase slows as the leakage grows uniformly. Panel (b) presents the corresponding precision improvement, which diminishes with higher background noise due to the increasing bias.

Additional sources of mode crosstalk include detector dark counts, signal background from incomplete extinction of the electro-optic modulator, and four-wave mixing (FWM) noise generated during the Raman interaction. In our experiment, we minimized the signal background using a Pockels cell and characterized both the dark counts and residual background, which were subtracted from the measured data. Furthermore, we applied appropriate detunings to the Raman transition to naturally suppress FWM noise, as described in Ref. [9]. Thus, the contribution of these sources is minimal.

7 Precise pulse carving

To improve our pulse carving system, we applied frequency response correction to our electronic system. In a linear time-invariant (LTI) system, where our electronic system can be approximated as such, the electric signal output $h(t)$ of the system to an arbitrary electric signal input function $x(t)$ can be approximately described by a linear response function $R(t-t')$ as

$$h(t) \approx \int_{-\infty}^{t} dt' R(t-t')x(t'). \tag{S15}$$

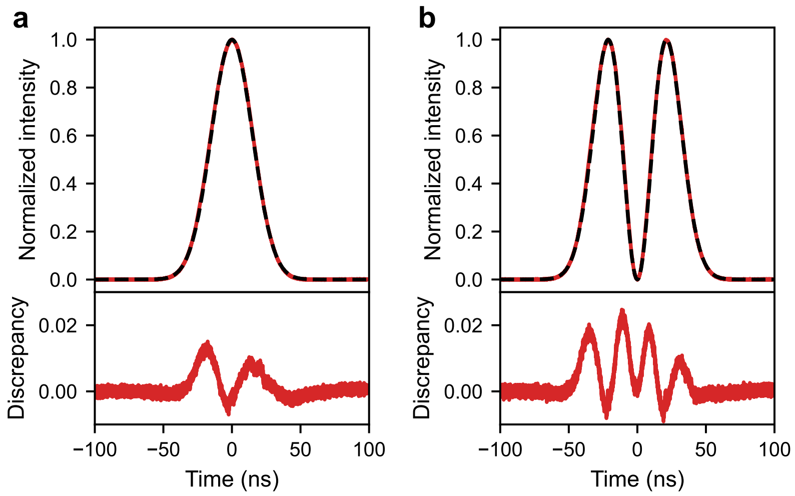

Fig. S6 Intensity profiles of experimental HG control pulses. (**a**) and (**b**) show the measured intensity profiles of the HG_0 and HG_1 control pulses, respectively. In the upper panels, the experimentally measured normalized profiles (solid red) are compared with the theoretical normalized HG profiles (dashed black). The lower panels display the discrepancies between the experimental and theoretical profiles.

In the frequency domain, the frequency response function $\tilde{R}(\omega)$ can be calculated as,

$$\tilde{R}(\omega) = \frac{\tilde{h}(\omega)}{\tilde{x}(\omega)}. \tag{S16}$$

Once the response function is known, a target signal $h'(t)$ can be precisely generated by using the response-corrected input signal $x'(t)$:

$$x'(t) = \mathcal{F}^{-1}\left[\frac{\mathcal{F}(h'(t))}{\tilde{R}(\omega)}\right]. \tag{S17}$$

where $\mathcal{F}$ denotes the Fourier transform and $\mathcal{F}^{-1}$ denotes the inverse Fourier transform.

To demonstrate our high-fidelity optical pulse carving system, we present the averaged and normalized intensity profiles of the HG_0 and HG_1 optical control pulses, as measured by a photodiode and an oscilloscope (1024 averages). Fig. S6(a) and (b) show the experimentally measured pulse shapes (red) compared against the theoretical intensity distributions (black dashed lines). The discrepancies between the theoretical and experimental data are plotted in the lower panels. The deviation between the measured and theoretical profiles is small, typically not exceeding 2% relative to the maximum.

References

[1] Nunn, J., Reim, K., Lee, K.C., Lorenz, V.O., Sussman, B.J., Walmsley, I.A., Jaksch, D.: Multimode memories in atomic ensembles. Physical Review Letters **101**, 260502 (2008)

[2] Klein, M., Hohensee, M., Nemiroski, A., Xiao, Y., Phillips, D.F., Walsworth, R.L.: Slow light in narrow paraffin-coated vapor cells. Applied Physics Letters **95**, 091102 (2009)

[3] Donohue, J.M., Ansari, V., Řeháček, J., Hradil, Z., Stoklasa, B., Paúr, M., Sánchez-Soto, L.L., Silberhorn, C.: Quantum-limited time-frequency estimation through mode-selective photon measurement. Physical Review Letters **121**, 090501 (2018)

[4] Mazelanik, M., Leszczyński, A., Parniak, M.: Optical-domain spectral super-resolution via a quantum-memory-based time-frequency processor. Nature Communications **13**(1) (2022)

[5] Lipka, M., Parniak, M.: Super-resolution of ultrafast pulses via spectral inversion. Optica **11**, 1226 (2024)

[6] Frehlich, R.: Effects of wind turbulence on coherent doppler lidar performance. Journal of Atmospheric and Oceanic Technology **14**, 54–75 (1997)

[7] McGill, M.J., Skinner, W.R., Irgang, T.D.: Validation of wind profiles measured with incoherent doppler lidar. Applied Optics **36**, 1928–1939 (1997)

[8] Huang, Z., Lupo, C., Kok, P.: Quantum-limited estimation of range and velocity. PRX Quantum **2**, 030303 (2021)

[9] Thomas, S.E., Hird, T.M., Munns, J.H.D., Brecht, B., Saunders, D.J., Nunn, J., Walmsley, I.A., Ledingham, P.M.: Raman quantum memory with built-in suppression of four-wave-mixing noise. Physical Review A **100**, 033801 (2019)
