# Supplementary material for: Super-resolving frequency measurement with mode-selective quantum memory
Source: Nat Sens. 2026 May 15;1(7):627–35. doi: 10.1038/s44460-026-00073-9 (PMC13368581; doi:10.1038/s44460-026-00073-9)
Supplement: Supplementary file 2 — Reporting Summary [file 44460_2026_73_MOESM2_ESM.pdf]

nature portfolio

Corresponding author(s): Aonan Zhang

Last updated by author(s): Mar 31, 2026

Reporting Summary

Nature Portfolio wishes to improve the reproducibility of the work that we publish. This form provides structure for consistency and transparency in reporting. For further information on Nature Portfolio policies, see our Editorial Policies and the Editorial Policy Checklist.

Statistics

For all statistical analyses, confirm that the following items are present in the figure legend, table legend, main text, or Methods section.

n/a	Confirmed	
☐	☒	The exact sample size (*n*) for each experimental group/condition, given as a discrete number and unit of measurement
☐	☒	A statement on whether measurements were taken from distinct samples or whether the same sample was measured repeatedly
☒	☐	The statistical test(s) used AND whether they are one- or two-sided<br>*Only common tests should be described solely by name; describe more complex techniques in the Methods section.*
☒	☐	A description of all covariates tested
☐	☒	A description of any assumptions or corrections, such as tests of normality and adjustment for multiple comparisons
☐	☒	A full description of the statistical parameters including central tendency (e.g. means) or other basic estimates (e.g. regression coefficient) AND variation (e.g. standard deviation) or associated estimates of uncertainty (e.g. confidence intervals)
☒	☐	For null hypothesis testing, the test statistic (e.g. *F*, *t*, *r*) with confidence intervals, effect sizes, degrees of freedom and *P* value noted<br>*Give P values as exact values whenever suitable.*
☒	☐	For Bayesian analysis, information on the choice of priors and Markov chain Monte Carlo settings
☒	☐	For hierarchical and complex designs, identification of the appropriate level for tests and full reporting of outcomes
☒	☐	Estimates of effect sizes (e.g. Cohen's *d*, Pearson's *r*), indicating how they were calculated

Our web collection on statistics for biologists contains articles on many of the points above.

Software and code

Policy information about availability of computer code

Data collection	The data were collected using superconducting nanowire single-photon detectors from Photon Spot, with photon counts registered using a Swabian Time Tagger 20. Data acquisition was implemented using Python commands from Swabian Time Tagger software (version 2.16). All data collection codes were written in Python 3.11.5. The code used for data collection is available via Zenodo at https://doi.org/10.5281/zenodo.19351042.
Data analysis	Data analysis was performed using Python 3.11.5. We utilized several key libraries, including NumPy, SciPy, Matplotlib, and h5py, to process and visualize the data. The code used for data analysis is available via Zenodo at https://doi.org/10.5281/zenodo.19351042.

For manuscripts utilizing custom algorithms or software that are central to the research but not yet described in published literature, software must be made available to editors and reviewers. We strongly encourage code deposition in a community repository (e.g. GitHub). See the Nature Portfolio guidelines for submitting code & software for further information.

Data

Policy information about availability of data

All manuscripts must include a data availability statement. This statement should provide the following information, where applicable:

- Accession codes, unique identifiers, or web links for publicly available datasets
- A description of any restrictions on data availability
- For clinical datasets or third party data, please ensure that the statement adheres to our policy

The data that support the findings of this study are included within the Article and its Supplementary Information. The processed photon count data files are available via Zenodo at https://doi.org/10.5281/zenodo.19351042.

Research involving human participants, their data, or biological material

Policy information about studies with human participants or human data. See also policy information about sex, gender (identity/presentation), and sexual orientation and race, ethnicity and racism.

Reporting on sex and gender	N/A.
Reporting on race, ethnicity, or other socially relevant groupings	N/A.
Population characteristics	N/A.
Recruitment	N/A.
Ethics oversight	N/A.

Note that full information on the approval of the study protocol must also be provided in the manuscript.

Field-specific reporting

Please select the one below that is the best fit for your research. If you are not sure, read the appropriate sections before making your selection.

☐ Life sciences ☐ Behavioural & social sciences ☒ Ecological, evolutionary & environmental sciences

For a reference copy of the document with all sections, see nature.com/documents/nr-reporting-summary-flat.pdf

Ecological, evolutionary & environmental sciences study design

All studies must disclose on these points even when the disclosure is negative.

Study description	Our research focuses on sensing the frequency separation of two spectral lines beyond the Rayleigh criterion, which may have applications in high-precision LiDAR systems. It is not directly related to ecology, evolutionary, or environmental sciences.
Research sample	The samples consist of incoherent mixtures of two laser pulses with equal intensities and preprogrammed frequency separations.
Sampling strategy	N/A. We uniformly distributed 20 frequency separations over the range from 0 to 1 pulse duration.
Data collection	The data were collected using superconducting nanowire single-photon detectors.
Timing and spatial scale	For each separation, measurements were performed with 2 control pulses and 4 phases, resulting in a total of 8 sets of measurements. Each set was collected for approximately 2 seconds with a pulse sequence length of 3 µs.
Data exclusions	No data were excluded from the analyses.
Reproducibility	The measurements were repeated multiple times, and the results showed good agreement.
Randomization	N/A. Bootstrapping was used in the analysis but not during data collection.
Blinding	N/A.

Did the study involve field work? ☐ Yes ☒ No

Reporting for specific materials, systems and methods

We require information from authors about some types of materials, experimental systems and methods used in many studies. Here, indicate whether each material, system or method listed is relevant to your study. If you are not sure if a list item applies to your research, read the appropriate section before selecting a response.

Materials & experimental systems

n/a	Involved in the study
☒	☐ Antibodies
☒	☐ Eukaryotic cell lines
☒	☐ Palaeontology and archaeology
☒	☐ Animals and other organisms
☒	☐ Clinical data
☒	☐ Dual use research of concern
☒	☐ Plants

Methods

n/a	Involved in the study
☒	☐ ChIP-seq
☒	☐ Flow cytometry
☒	☐ MRI-based neuroimaging

Plants

Seed stocks	N/A.
Novel plant genotypes	N/A.
Authentication	N/A.
