# Supplementary material for: Super-resolving frequency measurement with mode-selective quantum memory
Source: Nat Sens. 2026 May 15;1(7):627–35. doi: 10.1038/s44460-026-00073-9 (PMC13368581; doi:10.1038/s44460-026-00073-9)
Supplement: Supplementary file 3 — Peer Review File [file 44460_2026_73_MOESM3_ESM.pdf]

nature portfolio

Peer Review File

Super-resolving frequency measurement with mode-selective quantum memory

Corresponding Author: Dr Aonan Zhang

Version 0:

Decision Letter:

Dear Dr Zhang,

Your manuscript "Super-resolving frequency measurement with mode-selective quantum memory" has now been seen by 2 referees, and their comments are attached below. In the light of their advice, we have decided that we cannot offer to publish your manuscript in Nature Sensors.

From the reports, you will see that while they find your work of some potential interest and high technical quality, our referees raise concerns about the advance your findings represent over earlier work and the strength of the novel conclusions that can be drawn at this stage. We feel that these criticisms are sufficiently important as to prohibit publication of your work in Nature Sensors.

I am sorry that we cannot be more positive on this occasion, but hope that you find the referees' comments helpful when preparing your paper for resubmission elsewhere.

Although we cannot offer to publish your manuscript, we believe the editors at our sister journal, Nature Communications may find it interesting and recommend you transfer it there. To transfer your manuscript, please use our manuscript transfer portal. You will not have to re-supply manuscript metadata and files, unless you wish to make modifications, but please note that this link can only be used once and remains active until used. For more information, please see our manuscript transfer FAQ page.

Yours sincerely,

Dr. Olga Bubnova
Chief Editor
Nature Sensors

****

Reviewers' Comments:

Reviewer #1 (Remarks to the Author):

This paper demonstrate a super-resolving frequency measurement with mode-selective quantum memory in atomic vapor. While the technical results are solid and demonstrate a clear performance improvement in terms of enhancement factor over direct measurement, I have two major concerns regarding the novelty and significance of this work for Nature Sensors.

The core concept of utilizing a quantum memory in an atomic system to enhance frequency measurement resolution has been previously demonstrated. Specifically, a very similar physical principle and approach were reported in Nature Communications 13, 10.1038/s41467-022-28066-5 (2022). Although the current implementation in some technical details differs from the mentioned work, the overarching physical mechanism lacks sufficient novelty for a journal with the expected high-impact standing of Nature Sensors.

The journal Nature Sensors aims to cover more applied and engineering-focused research in sensing technology. A key metric for sensor applications is often the absolute performance. The authors strategically highlight the importance of their

enhanced precision relative to a direct intensity measurement baseline. However, the achieved absolute frequency resolution, while improved, does not surpass the state-of-the-art demonstrated ones in other works, including the one cited above. Consequently, the practical advancement and potential for immediate application impact seem limited. The performance, when viewed in the context of the absolute capabilities required for cutting-edge sensing applications, may not meet the criteria for publication in Nature Sensors.

In summary, while the work is technically sound and represents a competent engineering effort, the limited novelty and the lack of a demonstrable state-of-the-art absolute resolution make it, in my opinion, not suitable for publication in Nature Sensors. It might better fit to a more specialized journal focusing on quantum optics or atomic physics.

Reviewer #2 (Remarks to the Author):

The paper by Zhang et al. presents a practical super-resolved estimation protocol enabled by a quantum memory setup. The work explores Tsang's super-resolution idea in the time-frequency domain by enabling a mode-projective measurement using a shaped coupling field in the quantum memory.
In the paper, the authors first characterise the mode-sorting capability of the setup by measuring a cross-talk matrix, achieving impressive performance. This cross-talk later translates to an achievable super-resolved enhancement.
Next, the authors perform estimation by varying the frequency separation of two pulses. The estimation is studied broadly. I particularly value the proposed maximum-likelihood estimation method described in detail in the Supplement. Furthermore, the authors evaluate the estimation procedure for different photon numbers, also arriving at estimates of asymptotic performance. This nicely casts the work among other efforts, which are also broadly discussed in the final sections of the manuscript.
Overall, the manuscript presents a very high technical quality. The statistical analysis (very important in quantum estimation) is described in an excellent fashion. The details of the setup and the operation of the quantum memory are also given, ensuring - in my opinion - reproducibility.
I also think the paper presents an important advance and an exciting practical application of a quantum memory. It is not yet fully clear what particular engineering and sensing application this proof-of-principle method may be applied to, but I think that the transitional (fundamental to applications) character of the work warrants publication in Nature Sensors.

****

Version 1:

Decision Letter:

Dear Dr Zhang,

Thank you for submitting the revised version of your manuscript "Super-resolving frequency measurement with mode-selective quantum memory". It has now been seen again by our referees and their comments are attached below. In light of their advice, I am pleased to say that we can, in principle, offer to publish your article. First, however, we would like you to revise your paper to address the points made by the referees, and to make some editorial changes to ensure that it conforms to Nature Sensors format.

We are now performing checks on your paper and will send you a checklist detailing our editorial and formatting requirements in about a week. Please do not upload the final materials and make any revisions until you receive this additional information from us.

If you have any further questions please let me know.

Best wishes,

Dr. Olga Bubnova
Chief Editor
Nature Sensors

*****

ORCID

IMPORTANT: Non-corresponding authors do not have to link their ORCIDs but are encouraged to do so. Please note that it will not be possible to add/modify ORCIDs at proof. Thus, please let your co-authors know that if they wish to have their ORCID added to the paper they must follow the procedure described in the following link prior to acceptance:

https://www.springernature.com/gp/researchers/orcid/orcid-for-nature-research

Author names using non-Roman characters
Nature Portfolio journals can support presentation of author names using non-Roman characters in the HTML version of the article. If you wish to, please include author names in parentheses after the Roman-character spelling; see example online here. Currently supported scripts are: Arabic, Chinese, Cyrillic, Devanagari, Greek, Hebrew, Hangul, Japanese and Persian. You will be asked to verify the rendering is correct at proof stage.

****

Reviewer #1 (Remarks to the Author):

I thank the authors for their detailed and thoughtful rebuttal, as well as the revisions made to the manuscript. My original concerns centered on whether there is a genuine need to replace the GEM scheme with a Raman memory, a move that might appear to trade away the high resolution of narrowband techniques. In their response, the authors have addressed this question.
Through an identification of the problem to be solved (e.g., phase-decorrelated Doppler LiDAR), a feasibility analysis of their platform, and a quantitative comparison of key parameters against existing techniques, they have demonstrated that their approach is not a step backward, but a step sideways into an underserved domain with real-world application. I find these arguments persuasive, and I now recommend the manuscript for publication.

Reviewer #2 (Remarks to the Author):

I have studied the revised manuscript along with comments and responses. The authors have provided a comprehensive argumentation about the originality of their works. I agree with the authors that Raman mode projection provides distinct options for applications than previously demonstrated methods. In a way, it's important to notice that Raman-mode selectivity is, in a way, a simpler effect, indeed similar to the modal selection present in QPG. I think it is likely to be an important physical principle to be used in the narrowband regime, where rapid photon-counting-based distinction of frequency channels is required. The main limitation for most practical applications will be the requirement of the particular atom-resonant wavelength, but perhaps future work can improve tunability and/or be performed with broadened media.
Overall, I sustain my acceptance recommendation.

****

Open Access This Peer Review File is licensed under a Creative Commons Attribution 4.0 International License, which permits use, sharing, adaptation, distribution and reproduction in any medium or format, as long as you give appropriate credit to the original author(s) and the source, provide a link to the Creative Commons license, and indicate if changes were made.
In cases where reviewers are anonymous, credit should be given to 'Anonymous Referee' and the source.
The images or other third party material in this Peer Review File are included in the article's Creative Commons license, unless indicated otherwise in a credit line to the material. If material is not included in the article's Creative Commons license and your intended use is not permitted by statutory regulation or exceeds the permitted use, you will need to obtain permission directly from the copyright holder.
To view a copy of this license, visit https://creativecommons.org/licenses/by/4.0/

Response to reviewers' comments

Reviewer #1

Reviewer #1 acknowledged that our technical results are "solid" and demonstrate a "clear performance improvement" over direct measurements. The reviewer raised two major concerns regarding (1) the distinction between our work and a prior work based on gradient echo memory (GEM), and (2) the absolute frequency resolution in comparison with ultra-narrowband (kHz) systems. We provide a rebuttal to each point by clarifying that our approach relies on a fundamentally different, all-optical mapping physical mechanism that enables new sensing capabilities, and that the sensing resolution/precision is fundamentally related to the signal bandwidth. In the revised manuscript, we have added a comparison table, new data on programmability of mode filtering and analysis on sensing applications to solidify the novelty and significance of our work.

Comment 1: *This paper demonstrate a super-resolving frequency measurement with mode-selective quantum memory in atomic vapor. While the technical results are solid and demonstrate a clear performance improvement in terms of enhancement factor over direct measurement, I have two major concerns regarding the novelty and significance of this work for Nature Sensors.*

The core concept of utilizing a quantum memory in an atomic system to enhance frequency measurement resolution has been previously demonstrated. Specifically, a very similar physical principle and approach were reported in Nature Communications 13, 10.1038/s41467-022-28066-5 (2022). Although the current implementation in some technical details differs from the mentioned work, the overarching physical mechanism lacks sufficient novelty for a journal with the expected high-impact standing of Nature Sensors.

Reply 1: We respectfully disagree that our work uses a "very similar physical principle and approach" to Mazelanik et al. [*Nat. Commun.* 13, 691 (2022)]. While both works are under the broad label "atomic memory", the physical mechanisms are fundamentally different, leading to distinct sensing capabilities.

The general idea of achieving super-resolution via optimized mode projections is well established in quantum metrology and sensing, originally introduced by Tsang et al [*Phys. Rev. X* 6, 031033 (2016)]. The primary challenge for practical sensing applications is achieving high-fidelity, low-crosstalk mode projection experimentally. In this context, Mazelanik *et al.* employ spectral-spatial mapping with a gradient echo memory (GEM) to implement a fixed time-inversion interferometry. This physical principle relies on mapping frequency components to longitudinal spatial positions within an atomic ensemble, a process that requires laser-cooled atoms, precision magnetic field gradients, and spatial AC-Stark modulation. Consequently, this approach is physically constrained to a fixed symmetric/antisymmetric basis in an ultra-narrowband (kHz) regime.

In contrast, our scheme is fundamentally different in physical mechanisms, directly utilizing all-optical mapping in a stimulated Raman process to achieve arbitrary mode selectivity solely through temporal pulse shaping. This difference in physical mechanism leads to a different sensor architecture with distinct capabilities:

1. **Programmability:** While the spatial-mapping approach of GEM is hardwired for fixed symmetric/antisymmetric projections, our approach enables filtering of arbitrary user-defined modes by simply programming the electronic drive of the control laser pulse shaper. Benefiting from this, our platform is adaptable to diverse sensing tasks such as different line shapes and multi-parameter sensing - capabilities critical for the next generation of quantum sensors.

2. **Deployability:** Our approach removes the requirement for precise magnetic gradients, spatial AC-Stark modulation and laser-cooled atom handling, substantially reducing hardware complexity. This makes the platform a viable candidate for deployable, networked sensor nodes.

Taken together, these distinctions indicate that our work is not an incremental variation on the earlier work but a distinct, more capable physical route to time-frequency super-resolution. Our all-optical control approach is far more flexible and bandwidth-agnostic: the same physical principle can be applied to cold atoms [*Nat. Phys.* 8, 517 (2012), *Nat. Photon.* 9, 332 (2015)] in the kHz regime where we expect even higher precision enhancement or extended to high-frequency systems like hydrogen gas [*Phys. Rev. Lett.* 111, 083901 (2013)] or diamond [*Phys. Rev. Lett.* 114, 053602 (2015), *Nat. Commun.* **7**, 11200 (2016)] to reach THz bandwidths. Scaling the GEM platform to high-bandwidth regime (>1MHz) is technologically prohibitive under current implementations because increasing the spectral range requires steeper magnetic gradients, which reduce the spectral density of the optical depth and consequently the storage efficiency. Furthermore, the steep gradients compress the signal's spatial structure, making it highly susceptible to blurring from atomic motion and creating prohibitive hardware challenges due to the high inductance of the required gradient coils. To highlight these distinctions, we have

- Added **Section III** and **Table S1** in the Supplementary Information comparing different time-frequency super-resolution platforms in terms of physical principle, programmability, complexity, bandwidth, and precision enhancement.

TABLE S1. Comparison of time–frequency super-resolution sensor architectures

**Scheme**	**QPG**	**PuDTAI**	**SUSI**	**Raman**
**Principle**	Sum-frequency generation with shaped pulses	Time-inversion interferometry with spatial-spectral mapping	Spectral-domain inversion interferometry	Stimulated Raman scattering with pulse shaping
**Programmability**	Arbitrary mode	Fixed (symmetric/ antisymmetric)	Fixed (symmetric/ antisymmetric)	Arbitrary mode
**Storage & Conversion**	No	Storage	No	Yes
**Complexity**	Low (nonlinear waveguide, spatial-light modulator pulse shaping)	High (cold atoms, magnetic gradients, AC-Stark modulation)	Medium (interferometer stabilization, electro-optic lenses)	Low (warm vapor, electro-optical modulator pulse shaping)
**Bandwidth**	100s of GHz to THz	kHz to low MHz	10s of GHz to 100s of GHz	100s of kHz to low GHz
**Precision enhancement**	~ 24	~ 20	~ 2.13	~ 37

- Included new experimental data on the programmability of our platform (**Extended Data Fig. 1** in the revised manuscript, as shown below), demonstrating filtering of higher-order HG modes and their coherent superpositions.

A

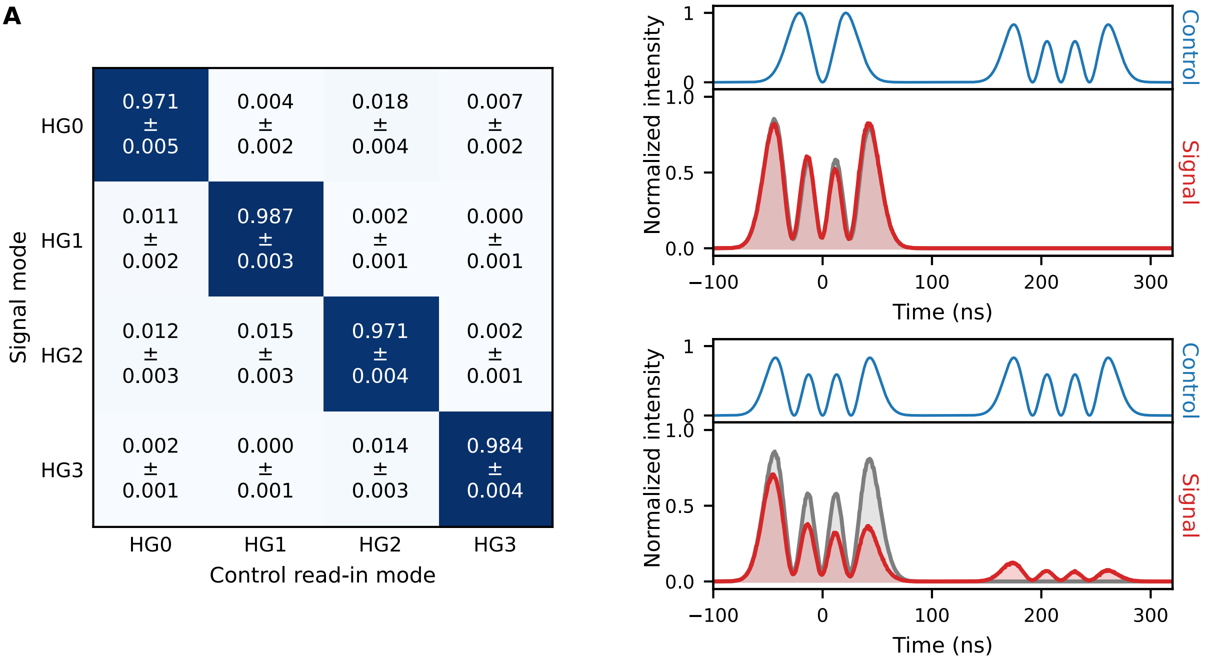

B

Signal	Control	Measured ratio	Ideal ratio
$(\mathrm{HG}_1+\mathrm{HG}_3)/\sqrt{2}$	$\mathrm{HG}_1, \mathrm{HG}_3$	1 : 1.02(14)	1 : 1
$(\mathrm{HG}_0+\mathrm{HG}_1+\mathrm{HG}_2)/\sqrt{3}$	$\mathrm{HG}_0, \mathrm{HG}_1, \mathrm{HG}_2$	1 : 1.37(25) : 0.99(21)	1 : 1 : 1
$(\mathrm{HG}_0+\mathrm{HG}_1 e^{i2\pi/3}+\mathrm{HG}_2 e^{i2\pi/3})/\sqrt{3}$	$\mathrm{HG}_0, \mathrm{HG}_1, \mathrm{HG}_2$	1 : 0.93(15) : 0.99(14)	1 : 1 : 1
HG_1	$\mathrm{HG}_1, (\mathrm{HG}_0+\mathrm{HG}_1 e^{i4\pi/3}+\mathrm{HG}_2)/\sqrt{3}$	3.08(28) :1	3 : 1
$(\mathrm{HG}_0+\mathrm{HG}_1+\mathrm{HG}_2 e^{i2\pi/3})/\sqrt{3}$	$\mathrm{HG}_0, (\mathrm{HG}_0+\mathrm{HG}_1+\mathrm{HG}_2 e^{i4\pi/3})/\sqrt{3}$	1 : 0.96(17)	1 : 1

Comment 2: *The journal Nature Sensors aims to cover more applied and engineering-focused research in sensing technology. A key metric for sensor applications is often the absolute performance. The authors strategically highlight the importance of their enhanced precision relative to a direct intensity measurement baseline. However, the achieved absolute frequency resolution, while improved, does not surpass the state-of-the-art demonstrated ones in other works, including the one cited above. Consequently, the practical advancement and potential for immediate application impact seem limited. The performance, when viewed in the context of the absolute capabilities required for cutting-edge sensing applications, may not meet the criteria for publication in Nature Sensors.*

Reply 2: We thank the reviewer for this comment, which allows us to clarify the relationship between bandwidth and resolution. In sensing contexts, resolution and precision are fundamentally tied to signal bandwidth and measurement time: for example, range resolution scales inversely with bandwidth ($\propto 1/B$) in radar/LIDAR and OCT, while frequency resolution scales with the bandwidth ($\propto B$) due to the fundamental time-frequency uncertainty principle [P. M. Woodward, Pergamon Press (1953), *Journal of the IEE*, 93(26), 429 (1946), *Science* 254, 1178 (1991)]. Comparing the absolute Hz resolution across sensors operating in different bandwidth regimes is analogous to comparing a GHz oscilloscope with an atomic clock, which serve distinct sensing domains. Specifically, narrowband sensors (kHz) are suitable for sensing static or slowly varying fields, but they are incapable of capturing fast transients and broadband signals. Our operation in the MHz-GHz regime fills a critical gap for dynamic sensing applications including LIDAR (resolving Doppler shifts in nanosecond

pulses) [*PRX Quantum* 2, 030303 (2021)], quantum-enhanced time/frequency transfer [*Phys. Rev. Lett.* 101, 123601 (2008)], frequency-encoded communication etc, which are inaccessible to slow kHz sensors.

The valid metric for sensing performance is the precision relative to the signal bandwidth. By this metric, our work (0.05× linewidth sensitivity and a 34-fold precision enhancement) significantly surpasses the state-of-the-art. The journal Nature Sensors "publishes fundamental, applied, and engineering research across the full spectrum of sensing technologies". Our work sits squarely within this fundamental-applied bridge, establishing a novel conclusion beyond prior work: all-optical control of Raman interactions enables a compact, room-temperature quantum memory to function as a high-precision and programmable time-frequency sensor, unlocking a dynamic regime (MHz-GHz) for broadband and reconfigurable sensing applications targeted by Nature Sensors.

In the revised version, we have clarified the relationship between resolution and signal bandwidth (page 3, line 116-118). We have also revised the text in Conclusions to highlight the above central advance of our work (page 12, line 524-531). In addition, we have added a discussion of sensing applications in the Conclusion section (page 11-12, line 506-523) and the Supplementary Information (**Section VII**), including an example in high-speed Doppler LiDAR, to show that our platform is directly beneficial to sensing applications operating in the MHz-GHz bandwidth regime.

Reviewer #2

Reviewer #2 praised the "very high technical quality" and "excellent" statistical analysis of the work and noted that the work represents an "important advance" that "warrants publication in Nature Sensors". They strongly recommended its publication along with a minor question on specific sensing applications. We thank the reviewer for their positive recommendation and address the question below.

Comment 1: *The work explores Tsang's super-resolution idea in the time-frequency domain by enabling a mode-projective measurement using a shaped coupling field in the quantum memory.*

In the paper, the authors first characterise the mode-sorting capability of the setup by measuring a cross-talk matrix, achieving impressive performance. This cross-talk later translates to an achievable super-resolved enhancement.

Next, the authors perform estimation by varying the frequency separation of two pulses. The estimation is studied broadly. I particularly value the proposed maximum-likelihood estimation method described in detail in the Supplement. Furthermore, the authors evaluate the estimation procedure for different photon numbers, also arriving at estimates of asymptotic performance. This nicely casts the work among other efforts, which are also broadly discussed in the final sections of the manuscript.

Overall, the manuscript presents a very high technical quality. The statistical analysis (very important in quantum estimation) is described in an excellent fashion. The details of the setup and the operation of the quantum memory are also given, ensuring - in my opinion - reproducibility.

I also think the paper presents an important advance and an exciting practical application of a quantum memory. It is not yet fully clear what particular engineering and sensing application this proof-of-principle method may be applied to, but I think that the transitional (fundamental to applications) character of the work warrants publication in Nature Sensors.

Reply 1: We are sincerely grateful to the reviewer for their highly positive assessment of our manuscript and the helpful feedback. Regarding sensing applications, we have expanded the Conclusions section to include a discussion of Doppler LiDAR as a representative example (page 11-12, line 506-523), complemented by a detailed explanation in the Supplementary Information (**Section VII**). We show how our broadband, incoherent, and super-resolving frequency measurement addresses limitations of conventional heterodyne and edge-filter techniques for frequency shift sensing, particularly under phase decorrelation and low-SNR conditions, illustrating the practical relevance of our platform for engineering and sensing applications.

Response to reviewers' comments

Reviewer #1
Remarks to the Author:
I thank the authors for their detailed and thoughtful rebuttal, as well as the revisions made to the manuscript. My original concerns centered on whether there is a genuine need to replace the GEM scheme with a Raman memory, a move that might appear to trade away the high resolution of narrowband techniques. In their response, the authors have addressed this question. Through an identification of the problem to be solved (e.g., phase-decorrelated Doppler LiDAR), a feasibility analysis of their platform, and a quantitative comparison of key parameters against existing techniques, they have demonstrated that their approach is not a step backward, but a step sideways into an underserved domain with real-world application. I find these arguments persuasive, and I now recommend the manuscript for publication.

Reply: We thank the reviewer for their positive assessment and for recognizing the value of our approach in sensing applications like Doppler LiDAR. We appreciate the recommendation for publication.

Reviewer #2
Remarks to the Author:
I have studied the revised manuscript along with comments and responses. The authors have provided a comprehensive argumentation about the originality of their works. I agree with the authors that Raman mode projection provides distinct options for applications than previously demonstrated methods. In a way, it's important to notice that Raman-mode selectivity is, in a way, a simpler effect, indeed similar to the modal selection present in QPG. I think it is likely to be an important physical principle to be used in the narrowband regime, where rapid photon-counting-based distinction of frequency channels is required. The main limitation for most practical applications will be the requirement of the particular atom-resonant wavelength, but perhaps future work can improve tunability and/or be performed with broadened media. Overall, I sustain my acceptance recommendation.

Reply: We thank the reviewer for their assessment regarding the physical principle of mode selectivity in our platform. The reviewer is correct that the current platform operates at specific atom-resonant wavelengths. Improving wavelength tunability is therefore an important direction for future work. We also note that, active sensing application like Doppler LiDAR do not impose strict wavelength constraints. We are grateful for the reviewer's positive evaluation and recommendation for publication.
